# Optimising neonatal fMRI data analysis: Design and validation of an extended dHCP preprocessing pipeline to characterise noxious-evoked brain activity in infants

**DOI:** 10.1016/j.neuroimage.2018.11.006

**Published:** 2019-02-01

**Authors:** Luke Baxter, Sean Fitzgibbon, Fiona Moultrie, Sezgi Goksan, Mark Jenkinson, Stephen Smith, Jesper Andersson, Eugene Duff, Rebeccah Slater

**Affiliations:** a Department of Paediatrics, University of Oxford, Oxford, United Kingdom; b FMRIB, Wellcome Centre for Integrative Neuroimaging, University of Oxford, Oxford, United Kingdom

**Keywords:** Developing human connectome project, Functional magnetic resonance imaging, Preprocessing, Haemodynamic response function, Neonate, Pain

## Abstract

The infant brain is unlike the adult brain, with considerable differences in morphological, neurodynamic, and haemodynamic features. As the majority of current MRI analysis tools were designed for use in adults, a primary objective of the Developing Human Connectome Project (dHCP) is to develop optimised methodological pipelines for the analysis of neonatal structural, resting state, and diffusion MRI data. Here, in an independent neonatal dataset we have extended and optimised the dHCP fMRI preprocessing pipeline for the analysis of stimulus-response fMRI data. We describe and validate this extended dHCP fMRI preprocessing pipeline to analyse changes in brain activity evoked following an acute noxious stimulus applied to the infant's foot. We compare the results obtained from this extended dHCP pipeline to results obtained from a typical FSL FEAT-based analysis pipeline, evaluating the pipelines' outputs using a wide range of tests. We demonstrate that a substantial increase in spatial specificity and sensitivity to signal can be attained with a bespoke neonatal preprocessing pipeline through optimised motion and distortion correction, ICA-based denoising, and haemodynamic modelling. The improved sensitivity and specificity, made possible with this extended dHCP pipeline, will be paramount in making further progress in our understanding of the development of sensory processing in the infant brain.

## Highlights

• The dHCP fMRI preprocessing pipeline is generalizable to stimulus-evoked datasets.
• Customised FIX denoising in infant fMRI data substantially improves data quality.
• The dHCP pipeline greatly improves spatial specificity and sensitivity to signal.
• Bespoke fMRI data analysis outperforms typical analytical methods for neonatal data.

## 1 Introduction

The infant brain is not a miniature replica of the adult brain. During early development, the composition, size, and morphology of the human brain changes rapidly (Dubois et al., 2014; Dubois and Dehaene-Lambertz, 2015), and neurodynamic and haemodynamic activity differs dramatically from that observed in adults (André et al., 2010; Arichi et al., 2012). Features such as the high water and low myelin content lead to a reduction in contrast and an inversion of MRI signal between tissue types (Paus et al., 2001), and data quality can be highly influenced by infant movement (Power et al., 2012; Reuter et al., 2015; Satterthwaite et al., 2012; Siegel et al., 2017; Yendiki et al., 2014). Despite these structural, functional, and behavioural differences, infant MRI studies often rely on data acquisition and analytical approaches that have been developed and refined to optimise spatial specificity and sensitivity to signal in adults.

Within the last decade, great strides have been made to change this by improving multiple aspects of neonatal fMRI data analysis and acquisition. The advantages of using infant-specific head coils and data acquisition parameters, such as echo time (TE), have been demonstrated (Goksan et al., 2017; Hughes et al., 2017), and novel stimulus-evoked experimental paradigms have been assessed (Cusack et al., 2015). Semi-automated independent component analysis (ICA)-based denoising (Salimi-Khorshidi et al., 2014) has previously been adapted for neonatal data (Ball et al., 2016); spatial smoothing extents have been scaled based on infant brain size (Gao et al., 2015); and haemodynamic response function (HRF) modelling has been optimised for a range of neonatal ages (Arichi et al., 2012). In addition, non-linear spatial normalisation tools, optimised for adult standard templates, have been adapted for use with neonate-specific templates (Goksan et al., 2015). This is not an exhaustive list, and those interested in the challenges and progress in neonatal fMRI are directed to recent reviews and references therein (Cusack et al., 2017; Mongerson et al., 2017) for further reading.

A major aim of the Developing Human Connectome Project (dHCP) is to understand human brain organisation in early life by modelling dynamic changes in structural and functional connectivity (www.developingconnectome.org). To achieve this goal, bespoke methodological approaches have been developed to optimise the analysis of neonatal structural, diffusion, and resting-state functional MRI data (Bastiani et al., 2018; Fitzgibbon et al., 2018; Makropoulos et al., 2018). The fMRI analysis pipeline has been designed to provide robust motion and distortion correction, optimised registration, improved structural templates, and automated artefact cleanup. However, to date this pipeline has not been applied to an independent dataset or used to quantify stimulus-evoked changes in blood oxygen level dependent (BOLD) activity. As such, its superiority over more traditional preprocessing approaches has not yet been demonstrated, and with its more extensive data manipulation and signal variance reduction, the risk of inadvertently removing signal-of-interest must be assessed.

Here, we assess these methodological innovations using a study of nociception in newborn infants. Accurate characterisation of noxious-evoked brain activity should provide invaluable insight into how pain perception develops in early life, but it requires a highly robust methodological approach. A mild noxious stimulus is repeatedly applied to the foot and noxious-evoked brain activity is recorded using fMRI. We extended the dHCP fMRI preprocessing pipeline to characterise the noxious-evoked BOLD activity, and compare the results to a typical analysis using FMRIB Software Library (FSL) FMRI Expert Analysis Tool (FEAT) procedures (Jenkinson et al., 2012) that we have previously used to study these responses in infants (Goksan et al., 2015). Noxious stimulation presents specific challenges such as the greater potential for stimulus-correlated motion artefacts due to reflexive activity, which can severely compromise signal quality. We aimed to assess the effects on data quality of bespoke motion and distortion preprocessing and robust spatial normalisation. In addition, we aimed to assess the effects of implementing semi-automated spatial ICA-based denoising, and of different choices of spatial smoothing and haemodynamic response function (HRF) modelling. Ultimately, we investigate the effect on spatial specificity and sensitivity to signal that can be attained with a bespoke preprocessing pipeline, optimised for neonatal stimulus-based fMRI data.

## 2 Material and methods

### 2.1 Infant demographics and experimental details

We recruited healthy term infants from the postnatal ward at the John Radcliffe Hospital (Oxford University Hospitals NHS Trust) for an MRI scan. Infants were eligible for inclusion in the study if they were greater than 37 weeks gestation and less than 10 postnatal days old, inpatients on the postnatal ward, never required admission to the neonatal unit, had no history of congenital conditions or neurological problems, and were clinically stable at the time of study. We scanned 15 term infants (8 male and 7 female) within the first postnatal week (mean postnatal age: 2.3 days; range 1–7 days). At the time of study, the mean gestational age (GA) of the infants was 39.3 weeks (range 37.1–42.7 weeks) and the mean birth weight was 3408 g (range 2235–4570 g). We obtained written informed consent from parents prior to study.

A clinical investigator transported infants to the Wellcome Centre for Integrative Neuroimaging (Oxford, UK), where infants were screened for metal, fed, and swaddled prior to scanning. Infants were fitted with ear-putty, ear-muffs (Minimuffs, Natus Medical Inc., Galway, Ireland), and ear-defenders (Em's 4 Bubs Baby Earmuffs, Em's 4 Kids, Brisbane, Australia), and placed on a vacuum-positioning mattress with extra soft padding around the head to restrict motion. We monitored heart rate and blood oxygen saturations throughout scanning (Fibre Optic Pulse Oximeter; Nonin Medical, Plymouth, Minnesota). We applied a 128 mN non-skin-breaking noxious stimulus (PinPrick Stimulator, MRC Systems) to the dorsum of the left foot 10 times, 1 s per trial, with a minimum inter-stimulus interval of 25 s. The stimuli were applied when the infants were naturally still, in order to minimise motion artefacts at the time of stimulus presentation, and were time-locked to the fMRI recording using Neurobehavioural Systems software (Presentation, www.neurobs.com). We obtained ethical approval for this study (National Research Ethics Service, REC reference: 12/SC/0447), and carried it out in accordance with the standards set by the Declaration of Helsinki and Good Clinical Practice guidelines.

All data were collected on the Siemens Prisma 3T with an adult 32 channel receive coil. The structural image acquisition was as follows: T2-weighted, TSE (factor 11), 150° flip angle, TE = 89 ms, TR = 14,740 ms, TA = 2 min 13 s, parallel imaging GRAPPA 3, 192 × 192 in-plane matrix size, 126 slices, 1 mm isotropic voxels. The fieldmap image acquisition was as follows: GRE, 2D FT readout, dual echo TE1/TE2 = 4.92/7.38 ms, TR = 550 ms, TA = 1 min 40 s, 46° flip angle, 90 × 90 in-plane matrix size, 56 slices, 2 mm isotropic voxels. The functional image acquisition was as follows: T2* BOLD-weighted, GRE, EPI readout, 70° flip angle, TE = 50 ms (Goksan et al., 2017), TR = 1300 ms, mean TA = 6 min (approx.), multiband 4 (Moeller et al., 2010; Xu et al., 2013), 90 × 90 in-plane matrix size, 56 slices, 2 mm isotropic voxels, with a single-band reference (SBref) image acquired at the start.

### 2.2 Introduction to the pipelines

#### 2.2.1 dHCP pipeline

**Fig. 1:**
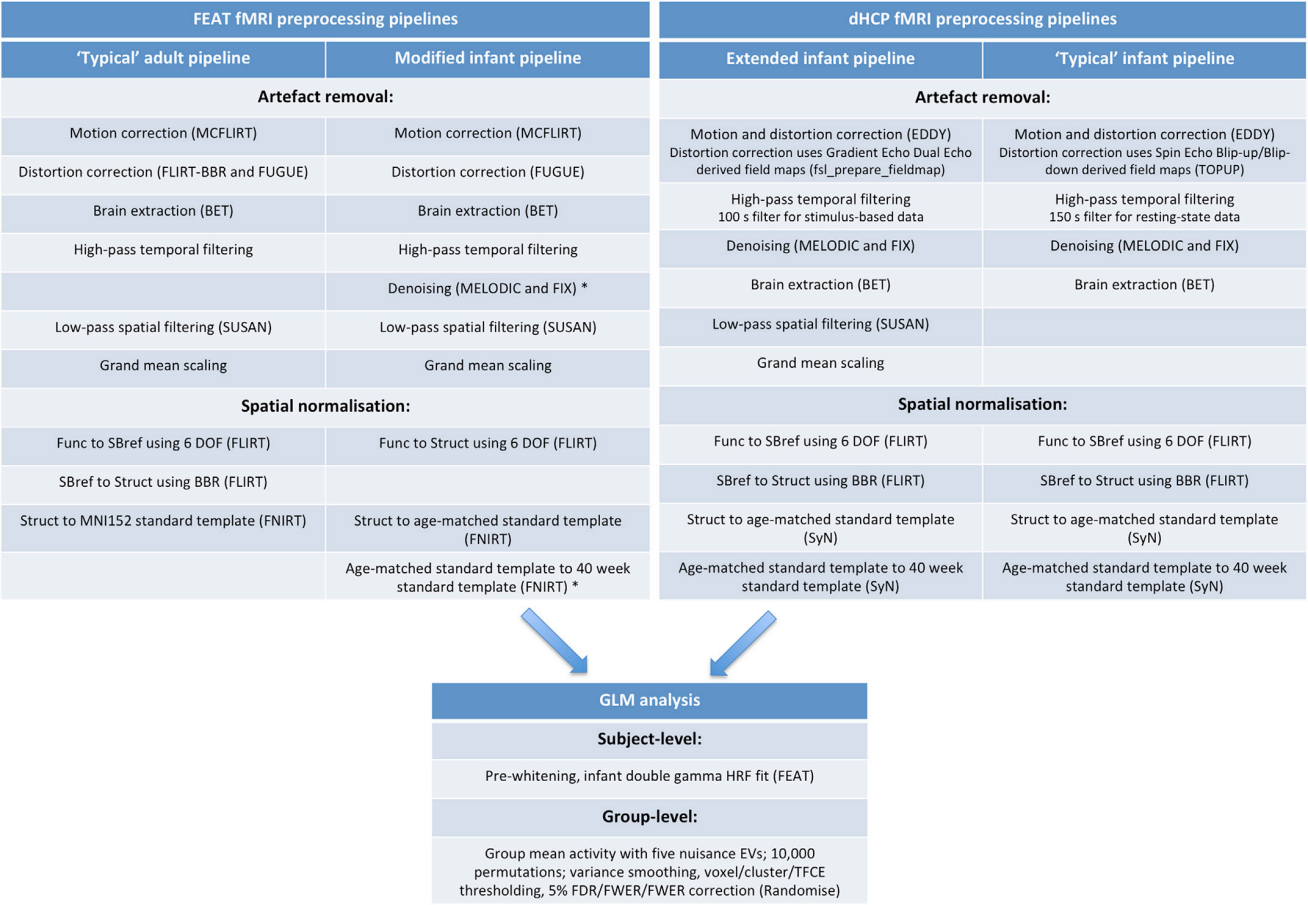
: fMRI analysis pipeline flowchart and comparison highlighting key pipeline differences. “FEAT fMRI preprocessing pipelines” box: the “’Typical’ adult pipeline” column is our hypothetical FEAT fMRI preprocessing pipeline using analysis tools and steps currently found in FEAT, appropriate for analysis of an adult multiband fMRI dataset; the “Modified infant pipeline” is our modification of this ‘typical’ FEAT pipeline for use in our infant multiband fMRI dataset (* = preprocessing steps performed partially or fully external to FEAT). “dHCP fMRI preprocessing pipelines box”: the “’Typical’ infant pipeline” column is a condensed description of some key analysis steps in the dHCP fMRI preprocessing pipeline; the “Extended infant pipeline” extends the dHCP pipeline for use with our stimulus-based fMRI dataset. “GLM analysis” box: this is a condensed description of the key GLM analysis steps performed at the subject- and group-level in this paper, and is common to the outputs of both our modified FEAT and extended dHCP fMRI preprocessing pipelines. See main text for expansion of abbreviations.

This pipeline was originally developed for, and tested on, dHCP resting-state data (Fitzgibbon et al., 2018; Fitzgibbon et al., in preparation), incorporating a variety of new and existing FSL tools, and other software to provide methods that are optimised for the characteristics of neonatal brains. The pipeline has not yet been formally published, but with the article in preparation, readers should refer to the Publications section of the dHCP website for further updates (www.developingconnectome.org). Here, we extended the pipeline, so that it could be applied to a stimulus-evoked dataset with different imaging parameters. We outline two key extensions here. First, we incorporated spatial smoothing to assess its effects on SNR and the effects of spatial normalisation misalignments. Second, the standard dHCP fMRI pipeline does not incorporate structural image preprocessing, because the dHCP structural images are first preprocessed in a separate structural preprocessing pipeline (Makropoulos et al., 2018). Therefore, the dHCP fMRI pipeline was extended to incorporate the MIRTK Draw-EM (Developing brain Region Annotation With Expectation- Maximization) neonatal pipeline v1.1 (Makropoulos et al., 2014), which is the neonatal segmentation tool that underpins the dHCP structural pipeline. This tool performs brain extraction, bias field correction, and tissue segmentation on neonatal T1/T2 images, which allows accurate and robust extraction of grey/white-matter boundaries for later registration steps. Throughout this paper, we refer to our extended dHCP preprocessing pipeline as ‘the dHCP pipeline’. See Fig. 1 for a schematic of the preprocessing steps in the typical and extended dHCP pipeline for functional images.

#### 2.2.2 FEAT pipeline

We compare the dHCP pipeline to a preprocessing pipeline using standard FSL FEAT tools (Jenkinson et al., 2012) with modifications for neonatal data, previously employed in Goksan et al. (2015) (see Fig. 1). All analysis steps are described in detail in sections below, but here we note three key modifications of the ‘typical’ FEAT pipeline for neonatal data. First, boundary based registration (BBR) (Greve and Fischl, 2009) was omitted, as the current default settings for FLIRT-BBR (FMRIB's Linear Image Registration Tool - BBR) in FEAT are hard-coded to adult specifications, which assume a specific direction of the intensity gradient across the white matter boundary, inappropriate for infants. The BBR parameters, which are used for distortion correction and functional-to-structural registration, cannot be altered within FEAT v6.00. Therefore, we used FEAT v5.98, which does not include BBR, for preprocessing in the FEAT pipeline (FEAT v6.00 was subsequently used for all subject-level GLM fitting in both the FEAT and dHCP pipelines, as outlined below in section *2.5.*). We assess the influence of BBR on the quality of spatial normalisation (as outlined in section *2.4.2.*), because this is one of the major differences between the pipelines' spatial normalisation approaches i.e. BBR is used in the dHCP pipeline but not used in our FEAT pipeline. Second, FIX (FMRIB's ICA-based Xnoiseifier) denoising (Griffanti et al., 2014; Salimi-Khorshidi et al., 2014), which is external to the FEAT pipeline, was included. FIX denoising is part of the standard dHCP pipeline, so its inclusion in our FEAT pipeline allowed for meaningful comparisons. We also explore the effect of omitting this step to demonstrate its central importance. Third, the final non-linear registration to standard templates was modified to include an extra registration step to accommodate the substantial changes in brain morphology during the neonatal period. Specifically, the structural T2 image was first non-linearly registered to a standard template corresponding to the infant's gestational week (Makropoulos et al., 2016), and then this age-matched template was non-linearly registered to a 40 week template, used as the global standard space. Throughout this paper, we refer to our modified instantiation of a ‘typical’ FEAT preprocessing pipeline as ‘the FEAT pipeline’. See Fig. 1 for a schematic of the preprocessing steps in the typical and modified FEAT pipeline for functional images.

### 2.3 Structural and field map image preprocessing

Both preprocessing pipelines required specific preparations of the structural and fieldmap images. For the dHCP pipeline, each subject's structural image was processed with the MIRTK Draw-EM neonatal pipeline v1.1 (as mentioned in section *2.2.1.*), and each fieldmap image was prepared using a modified version of fsl_prepare_fieldmap. For the FEAT pipeline, each subject's structural image was brain extracted using FSL's Brain Extraction Tool (BET) (Smith, 2002), with the optimal fractional intensity threshold and its vertical gradient parameters manually optimised per subject. Each subject's fieldmap image was prepared using fsl_prepare_fieldmap.

### 2.4 Functional image preprocessing

#### 2.4.1 Motion and distortion correction

Volume-to-volume motion correction was performed in the FEAT pipeline using MCFLIRT (Motion Correction FMRIB's Linear Image Registration Tool) (Jenkinson et al., 2002), which rigidly aligns volumes to the middle functional volume, correcting for between-volume motion. Distortion correction was performed using FUGUE (FMRIB's Utility for Geometrically Unwarping EPIs) (Smith et al., 2004), using static distortion correction. In the dHCP pipeline, volume-to-volume followed by slice-to-volume motion correction was implemented using EDDY (Andersson et al., 2017, 2016) to correct for between-volume motion and misaligned slices due to intra-volume motion. When applied to fMRI, EDDY treats each fMRI volume as a diffusion B0 image using a predictive model which assumes the contrast is identical across volumes. Subject head motion also causes changes in the susceptibility-induced field that result in changing distortions that cannot be adequately corrected using a static fieldmap method. EDDY corrects for this motion-by-susceptibility distortion by modelling the susceptibility-induced field as a continuous function of subject orientation to allow for the estimation of a unique susceptibility field for each volume (Andersson et al., 2018). Due to the differences in grey/white matter tissue contrast between our fieldmaps (gradient echo dual echo) and those in the dHCP dataset (spin echo blip-up/blip-down), we used a different (i.e. negative) BBR slope parameter for fieldmap registration to structural space prior to distortion correction (dHCP data: slope = 0.5; our data: slope = −0.5).

To assess the effects of these pipeline differences, we compared the DVARS motion metric and temporal signal-to-noise ratios (tSNR). From the general linear model (GLM) output of modelling the noxious stimuli events, we compared active voxel counts and t-statistics within specific grey matter regions-of- interest (ROIs) (see section *2.5.2.* for definition of ROIs). For all voxel counts and t-statistics comparisons, both the FEAT and dHCP preprocessing results were registered to standard space using FEAT-style registrations (detailed below) in order to disambiguate the effect of pipeline differences in motion and distortion correction from differences in spatial normalisation.

#### 2.4.2 Spatial normalisation

In the dHCP pipeline, the functional-to-structural-to-standard registration is a multi-step process. Using FSL's FLIRT (FMRIB's Linear Image Registration Tool), the functional reference volume was registered to the distortion-corrected SBref image using rigid-body 6 DOF, and the distortion-corrected SBref was then registered to the structural image using BBR (Greve and Fischl, 2009). The SBref image was distortion-corrected using FSL's FLIRT. The cerebral grey-matter/white-matter (GM/WM) boundary of the structural image was used for BBR. The registration between structural and standard space was performed using ANTs's SyN (Advanced Normalisation Tools's Symmetric image Normalisation method) (Avants et al., 2008), which is a diffeomorphic nonlinear registration. The structural image was first registered to the age-matched template, and the age-matched template was warped to the 40-week GA standard template by combining week-to-week non-linear warps from the age-matched template to the week 40 template. All transformations were combined into a single warp and applied once, to minimise data degradation due to interpolation.

Our modified FEAT pipeline follows a similar multi-step process to the dHCP pipeline, however the structural-to-standard registration steps are performed with FSL's FNIRT (FMRIB's Non-linear Image Registration Tool) (Andersson et al., 2007). Furthermore, as mentioned above in section *2.2.2.*, we did not use BBR in the functional-to-structural registration, as the current default settings for FLIRT-BBR in FEAT are hard-coded to adult tissue contrast specifications, which are inappropriate for infants, where the grey-white matter intensity gradient is inverted relative to adults.

For all spatial normalisation assessments, results were obtained using only the dHCP pipeline outputs in order to disambiguate spatial normalisation effects from other preprocessing effects, such as motion and distortion correction. We assessed the effects of these registration pipeline differences by comparing the alignment of the functional image in standard space and quantified normalized mutual information (NMI), which measures the statistical dependency between the two images. We also quantified the intensity gradient in the functional image across the cerebral GM-WM boundary of the standard template image. Using the GLM output of modelling the noxious stimuli events, we examined differences in active voxel counts and t-statistics within specific grey matter ROIs, and compared spatial specificity by comparing the proportion of significantly activated voxels incorrectly localised to white matter (see section *2.5.2.* for definition of grey and white matter ROIs).

#### 2.4.3 FIX denoising

The efficacy of FIX denoising (semi-automated sICA-based denoising) in neonatal fMRI data is not well documented. Considering that the noxious stimulus often elicits limb withdrawal reflexes, stimulus-correlated motion was expected (Hartley et al., 2015). Prior to FIX denoising in both pipelines, the data were high-pass temporally filtered using a 0.01 Hz (100 s period) cut-off, and data decomposed into independent components using FSL's MELODIC (Multivariate Exploratory Linear Optimised Decomposition into Independent Components) (Beckmann and Smith, 2004). FIX denoising was used in both our FEAT and dHCP pipelines.

**Fig. 2:**
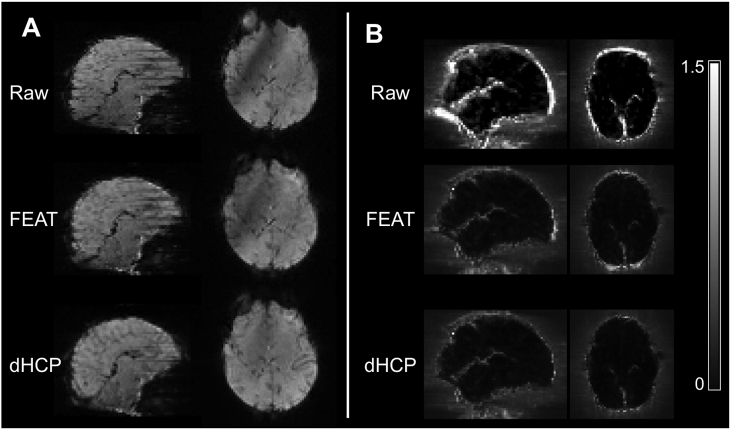
Comparison of the effects of motion correction and distortion correction between the FEAT and dHCP pipelines in representative subjects. (A) Comparison of volume-to-volume and slice-to-volume motion correction. Each row is the same volume from one subject before correction (Raw), after volume-to-volume motion correction used in the FEAT pipeline (FEAT), and after slice-to-volume motion correction used in the dHCP pipeline (dHCP). (B) Comparison of static distortion correction and estimated dynamic distortion correction. Each row is a standard deviation image as a proportion of the mean signal (i.e. ratio of temporal standard deviation to temporal mean) from one subject before correction (Raw), after static distortion correction used in the FEAT pipeline (FEAT), and after dynamic distortion correction used in the dHCP pipeline (dHCP). Improvements are seen predominantly at the brain surface perpendicular to the phase encode directions, especially in frontal and occipital polar regions.

**Fig. 3:**
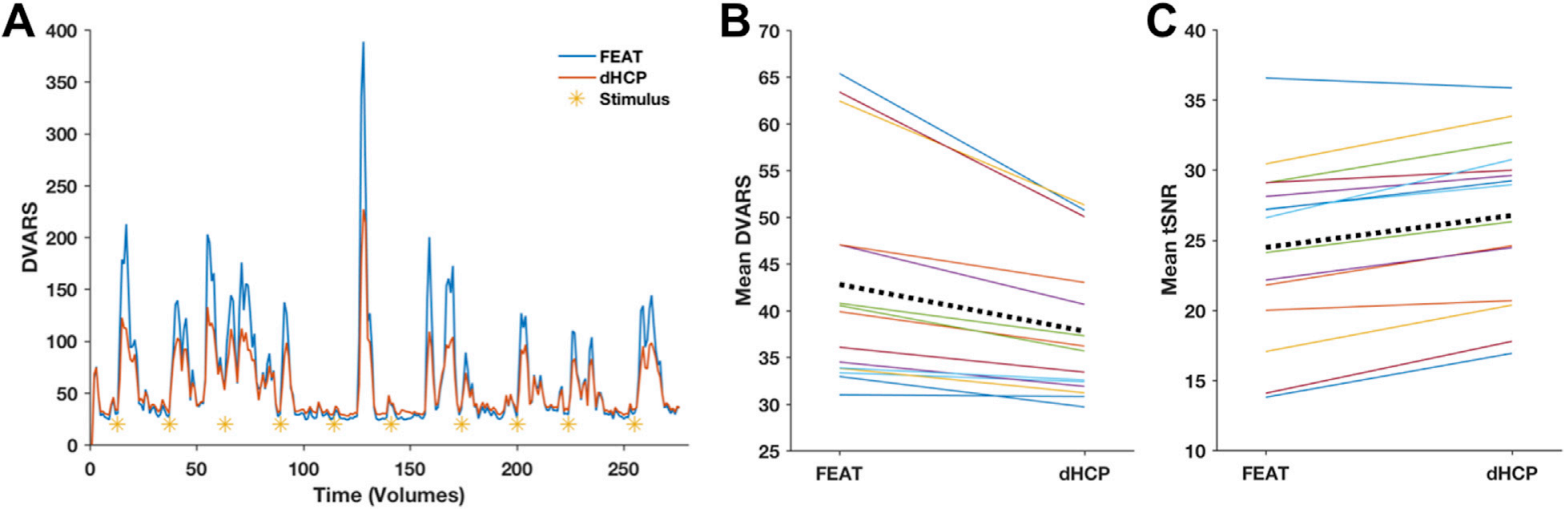
Comparison of effects of motion correction and distortion correction (MCDC) between FEAT and dHCP pipelines using measures of motion and signal-to-noise ratio. (A) Comparison of DVARS plots post-correction for a representative subject. The dHCP pipeline corrections result in lower mean DVARS metric across the entire session with greatest effects seen during large head motions. The yellow asterisks indicate the time of stimulus delivery, demonstrating the presence of stimulus-correlated motion in this subject. (B–C) Comparison of MCDC effects between FEAT and dHCP pipelines across all 15 subjects using DVARS, and tSNR. For each plot, solid coloured lines are individual subjects and the dotted black line is the group average. The dHCP MCDC results in lower DVARS and increased tSNR, indicating better artefact correction. The differences in DVARS and tSNR values between pipelines were statistically significant.

**Fig. 4:**
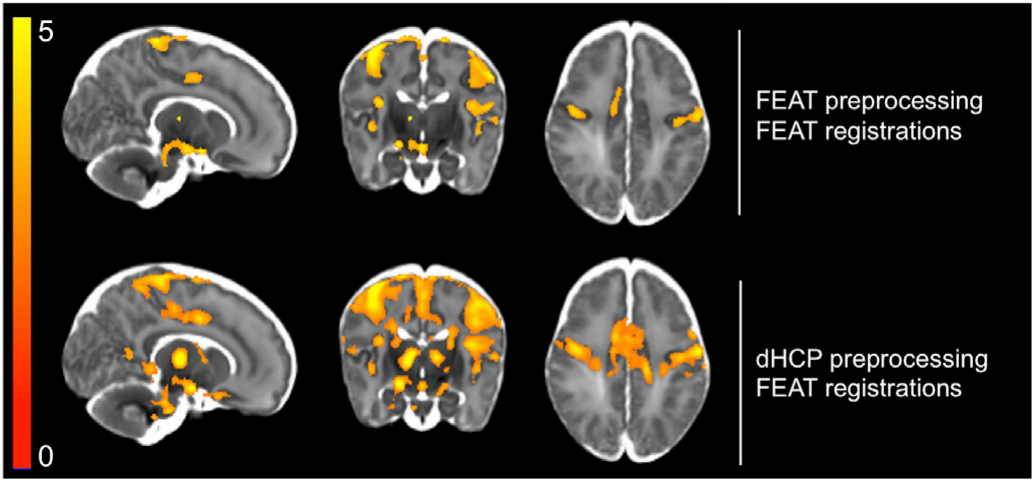
Comparison of motion correction and distortion correction (MCDC) between FEAT and dHCP pipelines using t-statistics from the GLM output for all 15 subjects. After thresholding the group activity maps (TFCE, default parameters, 5% FWER corrected), the dHCP pipeline resulted in greater sensitivity to signal in both cortical and subcortical regions. Using the dHCP pipeline, bilateral thalamic activity was detected. Using the FEAT pipeline, only contralateral thalamic activity was detected. Note, spatial smoothing is matched across FEAT and dHCP preprocessing pipelines.

**Fig. 5:**
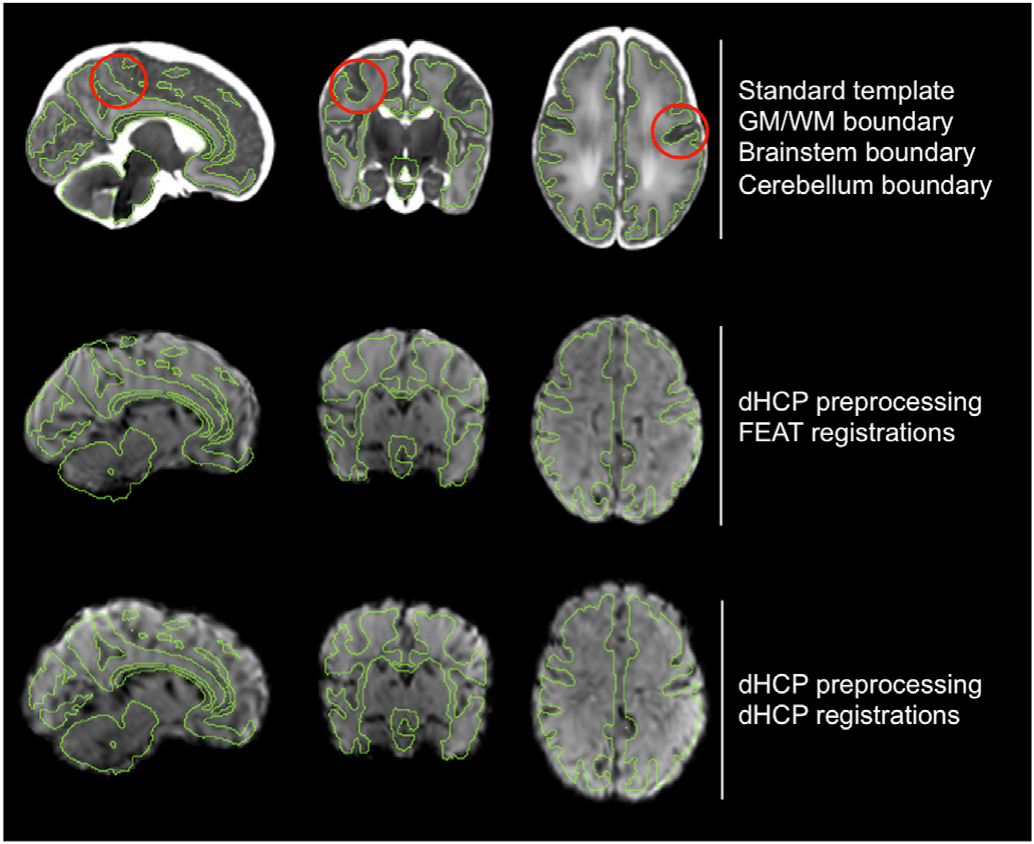
Comparison of the differences in spatial normalisation between the FEAT and dHCP pipelines in a representative subject. Top row: the week 40 GA standard template in grey-scale, with a tissue boundary overlaid in green to aid assessment of registrations. The boundary is taken from the same atlas as the standard template, and includes the GM-WM boundary in the cerebrum, as well as the outer boundary of the brainstem and cerebellum. Second row: the mean functional image registered to standard space using the FEAT pipeline registrations (FSL's FLIRT and FNIRT). Third row: the same mean functional image registered to standard space using the dHCP pipeline registrations (FSL's FLIRT-BBR and ANTs's SyN). In general, the FEAT registrations tend to incorrectly register the GM-CSF boundary of the functional image to the GM-WM boundary of the template, likely due to lack of BBR, and this is corrected in the dHCP result. Also, the cerebellum and brainstem are more accurately aligned with the template in the dHCP result. In this specific subject, several other improvements are visible in the dHCP results, with example regions in each view highlighted with a red circle.

**Fig. 6:**
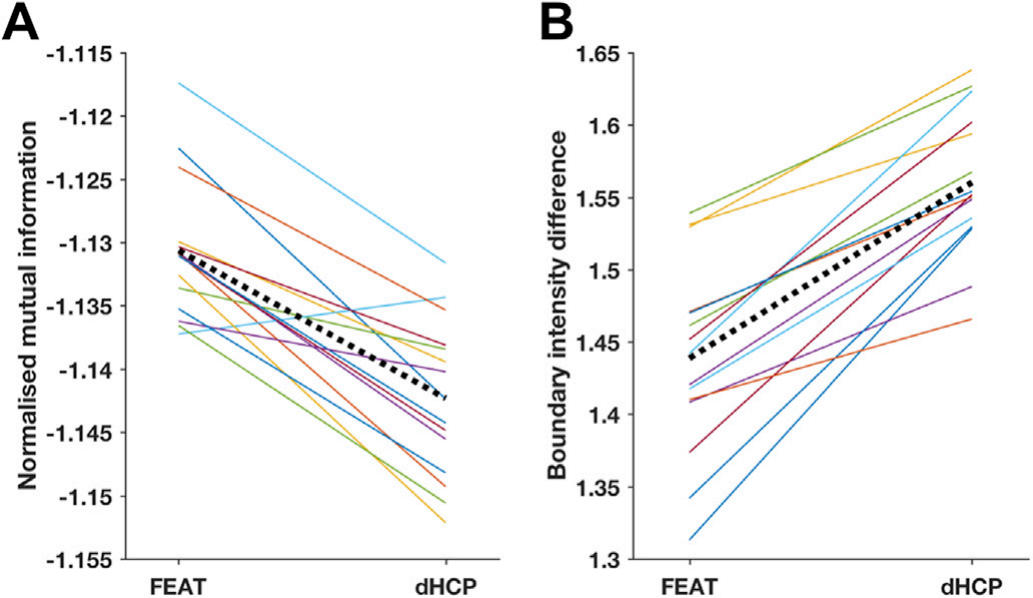
Comparison of spatial normalisation between FEAT and dHCP pipelines for all 15 subjects using (A) normalized mutual information and (B) boundary intensity difference. For each plot, solid coloured lines are individual subjects and the dotted black line is the group average. The dHCP spatial normalisation results in larger magnitudes for both alignment metrics, and these differences were statistically significant.

**Fig. 7:**
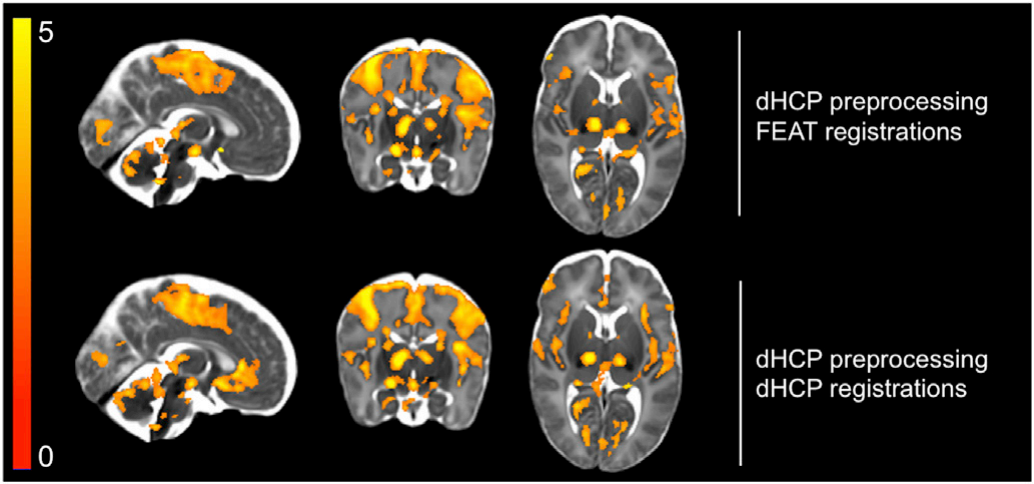
Comparison of spatial normalisation between FEAT and dHCP pipelines using t-statistics from the GLM output for all 15 subjects. The significant activity in the thresholded maps (TFCE, default parameters, 5% FWER corrected) more faithfully aligned with grey matter structures after applying the dHCP registration transformations. There was also better separation of activity in physically proximal brain regions that are separated by white matter, which should be devoid of activity e.g. the grey matter of the central sulcus and insular cortex.

**Fig. 8:**
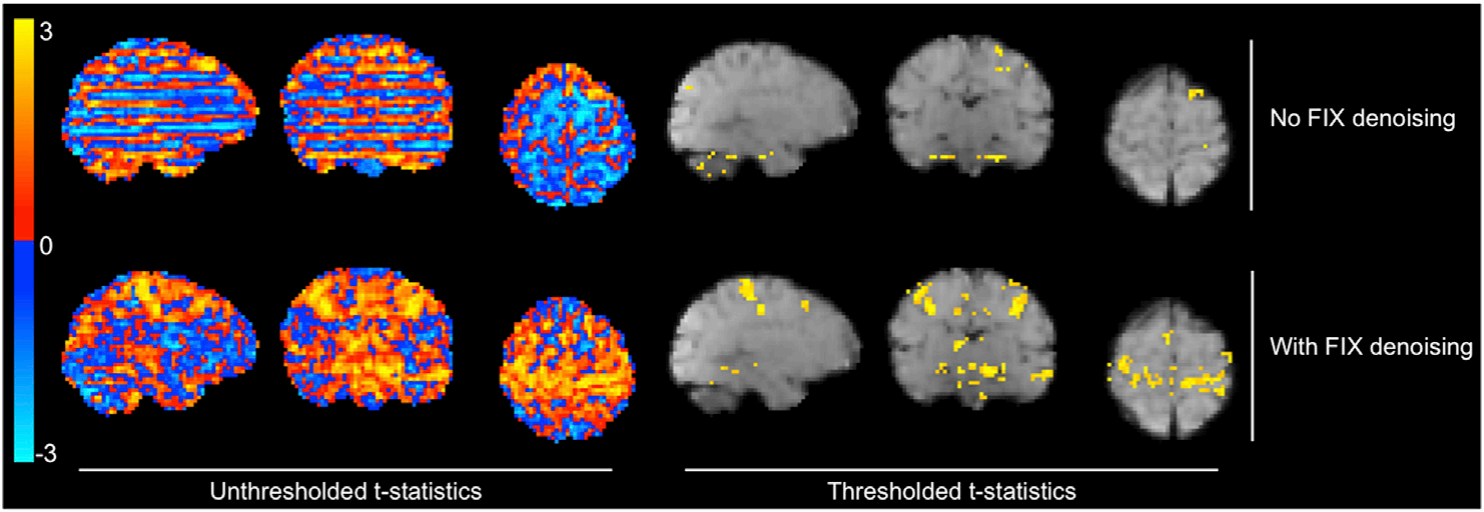
Visualization of the effect of FIX denoising using t-statistics from the GLM output for a representative subject with strong stimulus-correlated motion. Thresholding and correction for multiple comparisons of the GLM results was achieved using Gaussian random field theory cluster-based thresholding with a cluster-forming threshold of 2.3 and a 5% FWER correction. Top row: not using FIX denoising resulted in strong motion and striped multiband artefacts dominating the unthresholded t-statistic image, resulting in very poor sensitivity to signal in the thresholded image. Bottom row: using FIX denoising resulted in greatly reduced noise contamination of the unthresholded t-statistic image, and a significant improvement in sensitivity to signal in the thresholded image. Notable for this subject is the presence of stimulus-correlated motion; see Fig. 3A to see stimulus and head motion timings for this subject. FIX denoising allowed separation of sources of BOLD signal from motion artefacts successfully removing noise while retaining the signal of interest.

Noise components were manually labelled (Griffanti et al., 2017) and used to train FIX using the dHCP pipeline data only, because the FEAT pipeline cannot provide accurate tissue segmentations required by FIX for feature extraction. In the Supplementary material, we have provided eight examples of common ICA components identified in our data (Supp. Fig. 1, Fig. 2, Fig. 3, Fig. 4, Fig. 5, Fig. 6, Fig. 7, Fig. 8). To ensure accuracy and consistency of component labelling, we used FIX's built-in leave-one-out cross-validation testing. Any inaccuracies in component labellings identified here were inspected, components relabelled if necessary, and FIX was re-trained. This iterative process of training and manual inspection was considered complete when discrepancies between manual and FIX labellings were due solely to FIX mislabellings i.e. inspection of labelling discrepancies clearly demonstrated the manually assigned labels were correct. To ensure equally accurate FIX denoising between pipelines, the following approach was adopted. The trained FIX model was used in both pipelines initially only to label components. All components from both pipelines were then manually inspected, and misclassified components relabelled where necessary. Notably, the FIX model, which was trained on the dHCP data, worked equally well in the FEAT pipeline; the small number of misclassified components was comparable between pipelines, and no obvious difference in the nature of the components was discernible to the researchers. Once manual inspection of ICA components was complete, FIX was then used to remove these noise component time series and extended head motion parameter time series (24 motion time series) from the data.

We tested the effect of FIX denoising on the dHCP preprocessed data only, comparing the dHCP pipeline results with FIX denoising omitted to results with FIX denoising included. Using the GLM output of modelling the noxious stimuli events, we quantified the effect by looking at voxel counts and t-statistics within specific grey matter ROIs (see section *2.5.2.* for definition of ROIs). We used the dHCP preprocessing pipeline for this assessment to reduce the influence of noise and spatial normalisation misalignments on this analysis, as we had demonstrated that the dHCP pipeline was superior in terms of both motion and distortion correction, and spatial normalisation.

#### 2.4.4 Spatial smoothing

Spatial smoothing can improve SNR and reduce the effects of spatial normalisation misalignments (Lowe and Sorenson, 1997), at the expense of decreasing resolution to spatially localise activity. Using a filter with a full width at half maximum (FWHM) larger than an active region decreases sensitivity to activity in this region (Ball et al., 2012). This is important for neonatal fMRI analysis, considering the significantly smaller brain regions. We implemented spatial smoothing in both our FEAT and dHCP pipelines with FSL's SUSAN (Smoothing over Univalue Segment Assimilating Nucleus) (Smith and Brady, 1997), which uses neighbourhood voxel intensity information to limit voxel averaging to those voxels with similar intensities, preserving tissue structure by performing spatial smoothing within tissue type. The spatial filter must be larger than the voxel size, and we therefore used a minimal spatial filter extent equal to 1.5 times the voxel size (3 mm FWHM Gaussian kernel spatial filter). Finally, all data from both pipelines were grand mean scaled to have a spatiotemporal median value of 10,000 before doing the GLM analysis.

We also tested the effect of alternative spatial smoothing levels on the dHCP preprocessed data, assessing no spatial smoothing, minimal smoothing of 3 mm, and a 5 mm FWHM Gaussian kernel. This larger smoothing kernel was chosen as it has been previously used in studies of noxious-evoked brain activity in infants (Goksan et al., 2015) and is reasonably representative of the smoothing extent used in the adult literature. A review from 2012 (Carp, 2012) found an overwhelming majority (over 80%) of fMRI studies used a smoothing kernel equal to or greater than 5 mm FWHM, likely due to use of default settings (FEAT default: 5 mm FWHM; SPM default: 8 mm FWHM). Additionally, in the neonatal fMRI literature, examples of spatial filters larger than 5 mm are not uncommon in both resting-state (Mitra et al., 2017) and stimulus-based (Scheef et al., 2017) data analysis. We used the GLM output of modelling the noxious stimuli events to test the effects of spatial smoothing. We compared the active voxel counts and t-statistics within specific grey matter ROIs, and compared spatial specificity by comparing the proportion of significantly activated voxels incorrectly localised to white matter (see section *2.5.2.* for definition of grey and white matter ROIs).

### 2.5 GLM analysis

All subject-level GLM analysis for both the FEAT and dHCP pipelines was performed using FEAT v6.00. The stimulus event timings were convolved with a double gamma HRF tailored to neonates (see section 2.5.1*.* below for details). The model was temporally filtered using the same 0.01 Hz high-pass filter as applied to the data during preprocessing, and fitted to the data using FEAT (FSL version 5.0.10) with FILM (FMRIB's Improved Linear Model) prewhitening to correct for autocorrelations (Woolrich et al., 2001).

Two group-level analyses were performed: the first to estimate group activity for each pipeline separately using a whole-brain approach; the second to test if differences in t-statistics observed between the first group-level analyses were statistically significant using a region-constrained approach.

For the first group-level analysis approach, each subject's parameter estimate image was entered into a whole-brain analysis. The design matrix included five nuisance EVs for gestational age, postnatal age, gender, brain volume, and head motion (mean DVARS of entire raw time series). The mean stimulus-evoked positive response was estimated using permutation testing in FSL's Randomise (Winkler et al., 2014) with 10,000 permutations and 10 mm FWHM variance smoothing (Holmes et al., 1996) due to the relatively low degrees of freedom. Thresholded group activity maps, corrected for multiple comparisons, were generated using three separate approaches: first, voxel-based thresholding with a 5% false discovery rate (FDR) correction (using family-wise error rate correction for voxel-based whole brain analysis was too conservative, resulting in many empty thresholded maps); second, cluster-mass-based thresholding (Bullmore et al., 1999) with a cluster-defining threshold of 2.3 and a 5% family-wise error rate (FWER) correction; third, threshold-free cluster enhancement (TFCE) (Smith and Nichols, 2009) thresholding with default parameters and a 5% FWER correction. Because each thresholding approach has strengths and shortcomings, quantitative comparisons are reported for all three approaches to facilitate interpretation.

For the second group-level analysis approach, a difference-in-t-statistic image was generated per subject per pipeline comparison and entered into a region-constrained voxel-wise analysis (see section 2.5.2*.* below for details of ROI and constrained region definition). Due to the constrained region being composed of several discrete non-contiguous ROIs, thresholding approaches that use neighbourhood spatial information, such as cluster-based and TFCE-based thresholding, were not valid. Thus, we used voxel-based thresholding with a 5% FWER correction for these cross-pipeline region-constrained analyses. Similar to the first group-level analysis, we used Randomise with 10,000 permutations, variance smoothing, and controlled for the same nuisance variables.

#### 2.5.1 HRF modelling

Compared to adults, the BOLD response of neonates has a smaller amplitude, longer latency to peak, larger undershoot relative to initial rise, and longer latency to return to baseline (Arichi et al., 2012; Colonnese et al., 2008). Thus neonate-specific HRF models are necessary to accurately model the response to a stimulus. To test the effect of different HRF models, we used the dHCP pipeline results with three different HRFs of increasing complexity. We examined a single gamma (without undershoot; abbreviated as SG), a double gamma (with initial rise and subsequent undershoot; abbreviated as DG), and a three-basis-function model developed using FSL's FLOBS (abbreviated as FLOBS) (FMRIB's Linear Optimal Basis Sets) (Woolrich et al., 2004). Both the SG and DG HRF models were generated in-house using the neonate-appropriate parameterisations (based on (Arichi et al., 2012)).

To examine the effects of these different HRF models on GLM output, we generated a size-statistic image and its associated t-statistic image per subject. For the SG and DG HRFs, the size-statistic image was the typical parameter estimate image automatically generated by FEAT. For the FLOBS HRF, we chose the size statistic to be the 2-norm of the fitted FLOBS model, taking the sign of the parameter estimate from the first basis function. As described above (section *2.5.*), subjects' size-statistic (parameter estimate) images were entered into our first group-level analysis approach; subjects’ t-statistic images were used to generate difference-in-t-statistic images and entered into our second group-level analysis approach. We examined the effects of these different HRF models on GLM output by comparing active voxel counts and t-statistics within specific grey matter ROIs (see section *2.5.2.* for definition of ROIs).

To understand how our three HRF models fit to the data, we examined peristimulus time plots in two ROIs: the postcentral gyrus, due to the robust signal detection using all HRF models, and the thalamus, due to the large variability in sensitivity between models. For each subject, we extracted one time series per ROI by averaging across all trials, and extracting the mean time series across all ROI voxels. We used a time window of 20 vol (26 s), from the point of stimulus delivery, due to our minimum inter-stimulus interval being 25 s. In addition to subject-level peristimulus time plots, we compared group average plots. For the raw data plots, we used the Woody average to correct for artefactual jitter in the time series; for the HRF plots, we used a simple average. We compared the stimulus responses in the raw data to the HRF estimates by examining latency from baseline-to-peak and upshoot-to-undershoot amplitude ratio. To extract robust values from the raw data, we fitted a double gamma function to the raw data group average, which fitted accurately. The baseline-to-peak and upshoot-to-undershoot ratio for the raw data were extracted from the fitted double gamma function. All peristimulus time plot analyses were performed using standard FSL and MATLAB tools.

#### 2.5.2 ROI and region-constrained analyses

We defined four grey matter and one white matter ROIs as follows. In an independent dataset of 15 term subjects (Goksan et al., 2018), we used the FEAT pipeline to generate an activity map in response to the 128 mN noxious stimulus, which was thresholded (TFCE default parameters, 5% FWER) and binarised to generate an activity mask. We defined four bilateral anatomical regions using the infant standard brain atlas (Makropoulos et al., 2016): the thalamus, insula, postcentral gyrus (PoCG), and anterior cingulate cortex (ACC). The thalamus, insula, and ACC regions were defined directly from the atlas, whereas the postcentral gyrus was defined manually as follows. The anterior boundary with the precentral gyrus and the medial boundary were automatically defined using the atlas. The posterior boundary was manually selected as the fundus of the postcentral sulcus and the lateral boundary with the opercular cortex was manually defined using the lateral sulcus. We then masked the anatomical masks with the activity mask, in order to define four discrete, functionally active, grey matter regions of interest (ROIs). The thalamus ROI includes regions of the thalamus involved in relaying incoming noxious stimulus information to the cortex. The postcentral gyrus ROI includes primary somatosensory cortex, area S1. The insula and ACC ROIs include subregions of these structures involved in processing noxious stimulus information. We also generated a fifth white matter ROI using the atlas 40-week white matter mask, which we used to identify activity incorrectly localised to white matter during the spatial normalisation and spatial smoothing comparisons.

To compare the group GLM t-statistic results between pipelines, we used the above four bilateral grey matter ROIs in an ROI analysis approach. First, to generate the group-level t-statistic images, each subject's effect size image was entered into the group-level whole-brain analysis as described above (section *2.5.*). Then, we examined the effect of pipeline differences on group-level GLM statistics by comparing mean and peak t-statistics extracted from each of the grey matter ROIs. Here, we follow the rationale of Smith and colleagues that, at the group-level, it is reasonable to assume that larger t-statistics are better due to reduced noise variance originating from imperfect analysis methods (Smith et al., 2005). To test if these observed differences were statistically significant, we generated difference-in-t-statistic images per subject per pipeline comparison and entered these into group-level region-constrained analyses. The region to which the analyses were constrained was a reduced infant ‘pain network’ defined by combining the above four grey matter ROIs.

## 3 Results

### 3.1 Assessment of motion correction and distortion correction (MCDC)

The dHCP pipeline corrected slice-to-volume and motion-by-susceptibility artefacts that were not corrected by the FEAT pipeline (Fig. 2). The dHCP dynamic fieldmap approach provided greatest noise reduction in frontal and occipital polar regions. We quantified motion-related noise remaining after MCDC using DVARS, and by comparing the tSNR (Fig. 3). At the single subject level, the dHCP MCDC resulted in a greater reduction in motion related signal variance, especially during large movements (Fig. 3A). At the group level, the dHCP MCDC pipeline significantly reduced the mean DVARS values (Wilcoxon signed-rank test, α = 0.05, p = 1.8*10^−4^) and increased the mean tSNR values (Wilcoxon signed-rank test, α = 0.05, p = 6.1*10^−5^) across subjects (Fig. 3B and C). Note that the change in tSNR should be interpreted with caution, and in combination with the other measures, as real signal fluctuations in the data can be included in the ‘noise’.

The dHCP MCDC resulted in a modest increase in the mean group-level t-statistic in all grey matter ROIs (Table 1). Comparing the number of significantly active voxels, all three thresholding approaches demonstrated increased number of active voxels using the dHCP pipeline (Table 1). Using the thresholded activity maps to qualitatively compare pipelines, the increased significant activity was seen in both cortical and subcortical regions, with greatest improvements in the thalamus (Fig. 4). Together, these results suggest the dHCP MCDC pipeline increased sensitivity to signal by reducing noise variance. Comparing the pipeline differences in subject-level t-statistics, the changes in these statistics were not statistically significant.

**Table 1:** : Comparison of motion correction and distortion correction (MCDC) between FEAT and dHCP pipelines using t-statistics from the GLM output and significantly activated voxel counts for all 15 subjects. T-statistics: using the dHCP pipeline, all grey matter ROIs had an increase in mean t-statistic. The maximum t-statistic (in parentheses) increased in all regions using the dHCP pipeline, except the PoCG. # active voxels: the dHCP pipeline also resulted in an increase in the number of significantly active voxels using all three thresholding approaches. Voxel is FDR corrected; cluster and TFCE are FWER corrected. ACC = anterior cingulate cortex; PoCG = postcentral gyrus; TFCE = threshold free cluster enhancement.

		FEAT	dHCP
T-statistics	ACC	1.529 (3.518)	2.012 (4.581)
	Insula	2.651 (5.244)	2.682 (5.422)
	PoCG	2.822 (6.568)	3.131 (6.123)
	Thalamus	2.168 (6.197)	2.542 (6.368)

# active voxels	Voxel	0	41,492
	Cluster	60,175	62,224
	TFCE	16,823	53,926

### 3.2 Assessment of spatial normalisation

Improvements in spatial normalisation were apparent in individual subjects when using the dHCP pipeline (Fig. 5). Greatest improvements were visible at the brain/non-brain surface. The FEAT pipeline registrations frequently incorrectly aligned the functional image cortical GM-CSF boundary with the template image cortical GM-WM boundary, unlike the dHCP pipeline. The dHCP registrations also produced marked improvements in cerebellum and brainstem alignment. Other non-surface improvements were visible but less consistent across subjects. To quantify these differences in spatial normalisation, we compared the alignment between the functional image in standard space and the standard template image using normalized mutual information (NMI), and the intensity difference of the functional image across the GM-WM cerebral boundary of the standard template i.e. the boundary intensity difference (Fig. 6). Using both metrics, the dHCP registrations resulted in a statistically significant improvement in spatial normalisation, (Wilcoxon signed-rank test, α = 0.05, p = 1.2*10^−4^ for NMI, p = 6.1*10^−5^ for boundary intensity difference).

The dHCP spatial normalisation resulted in a modest increase in the mean group-level t-statistic in all grey matter ROIs, except the PoCG (Table 2). Comparing the number of significantly active voxels and the proportion of significant activity incorrectly localised to white matter, all three thresholding approaches demonstrated increased number of active voxels and decreased proportion of mislocalised activity using the dHCP pipeline (Table 2). Using the thresholded activity maps to qualitatively compare pipelines, the dHCP pipeline provided greater spatial specificity, limiting the regions of significant activity to the grey matter more accurately than the FEAT pipeline (Fig. 7). Together, these results suggest the dHCP spatial normalisation pipeline increased spatial specificity and sensitivity to signal by reducing subject-template and subject-subject misalignment errors. The subject-level t-statistics between pipelines were however not statistically significantly different.

**Table 2:** Comparison of spatial normalisation between FEAT and dHCP pipelines using t-statistics from the GLM output and significantly activated voxel counts for all 15 subjects. T-statistics: using the dHCP pipeline, there was an increase in the mean t-statistic in all grey matter ROIs, except the PoCG (maximum t-statistic in parentheses). # active voxels: the dHCP pipeline resulted in greater sensitivity to signal, measured as increased number of active voxels using all three thresholding approaches. % white matter: the dHCP pipeline resulted in greater spatial specificity, measured as decreased percent of active voxels mislocalised to white matter using all three thresholding approaches. Voxel is FDR corrected; cluster and TFCE are FWER corrected. ACC = anterior cingulate cortex; PoCG = postcentral gyrus; TFCE = threshold free cluster enhancement.

		FEAT	dHCP
T-statistics	ACC	2.012 (4.581)	2.104 (4.030)
	Insula	2.682 (5.422)	2.977 (5.224)
	PoCG	3.131 (6.123)	2.959 (6.987)
	Thalamus	2.542 (6.368)	2.565 (6.797)

# active voxels	Voxel	41,492	66,984
	Cluster	62,224	82,837
	TFCE	53,926	66,756

% white matter	Voxel	20.835	17.598
	Cluster	9.882	5.400
	TFCE	8.504	4.687

### 3.3 Assessment of FIX denoising

**Fig. 9:**
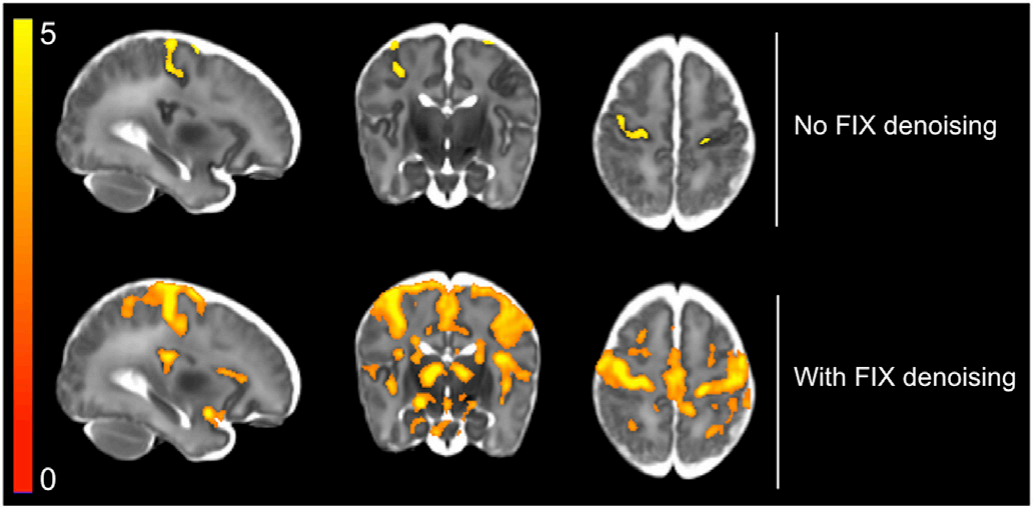
Comparison of the effects of FIX denoising on t-statistics from the GLM output for all 15 subjects. Comparing thresholded group activity maps (TFCE, default parameters, 5% FWER corrected), there is a dramatic increase in sensitivity to signal, in both cortical and subcortical regions, when using FIX denoising compared to no FIX denoising. Note, this FIX denoising comparison was assessed using dHCP pipeline outputs only.

We custom-trained FIX using the 15 subjects’ data from the dHCP pipeline, as described in the methods. Using the inbuilt leave-one-out cross-validation, we assessed its accuracy for automatic denoising of new subjects to have a median True Positive Rate (TPR; percent of signal components correctly identified as signal) of 100%, a median True Negative Rate (TNR; percent of noise components correctly identified as noise) of 95%, and an overall measure of median accuracy of 98.6% (accuracy defined in FIX as (3*TPR + TNR)/4) (see Salimi-Khorshidi et al. (2014) for details). Using the dHCP pipeline, the effects of FIX denoising can be seen at both the subject-level (Fig. 8) and group-level (Fig. 9), demonstrating a dramatic improvement in sensitivity to signal. Particularly in subjects with strong stimulus-correlated motion, BOLD and motion signal sources were clearly separated using ICA, and noise was successfully removed from the data using FIX (see Fig. 8). White matter and CSF signal sources were readily visible as ICA components, negating the need to manually extract signal time courses from these regions using ROIs. Removing these signal sources using the ICA approach allowed us to avoid the partial volume risk inherent to the ROI approach i.e. inadvertently including grey matter in the white matter and CSF ROIs. This risk was substantially greater in our infant dataset than in typical adult datasets due to the smaller brain and ventricle size, resulting in overall poorer voxel-wise tissue type resolution. An additional benefit of the FIX approach to ICA denoising was its semi-automated nature.

Including FIX denoising in the preprocessing pipeline resulted in an increase in the mean group-level t-statistic in all grey matter ROIs (Table 3). Comparing the number of significantly active voxels, all three thresholding approaches demonstrated dramatically increased number of active voxels using FIX denoising (Table 3). Using the thresholded group activity maps to qualitatively compare pipelines, an increase in significant activity was seen globally (Fig. 9). Together, these results suggest FIX denoising dramatically increases sensitivity to signal by reducing noise variance. Comparing the differences in subject-level t-statistics, these differences were statistically significant (voxel-based thresholding, 5% FWER corrected), localised to all grey matter ROIs, except the ACC.

**Table 3:** Comparison of the effects of FIX denoising on t-statistics from the GLM output and significantly active voxel counts for all 15 subjects. T-statistics: using FIX denoising resulted in an increase in the mean t-statistic in all grey matter ROIs (maximum t-statistic in parentheses). * = ROIs in which FIX denoising resulted in statistically significant increases in t-statistics (voxel-based thresholding, 5% FWER corrected). # active voxels: using FIX denoising also resulted in an increase in the number of significantly active voxels using all three thresholding approaches. Voxel is FDR corrected; cluster and TFCE are FWER corrected. ACC = anterior cingulate cortex; PoCG = postcentral gyrus; TFCE = threshold free cluster enhancement.

		No FIX	With FIX
T-statistics	ACC	2.065 (4.822)	2.104 (4.030)
	Insula	2.199 (4.839)	2.977* (5.224)
	PoCG	2.249 (5.329)	2.959* (6.987)
	Thalamus	1.527 (7.387)	2.565* (6.797)

# active voxels	Voxel	0	66,984
	Cluster	30,837	82,837
	TFCE	1311	66,756

### 3.4 Assessment of spatial smoothing

Changing spatial smoothing extent shifted the balance between sensitivity to signal and spatial specificity. Comparing the ROI analysis results, we observed a consistent decrease in maximum t-statistic across all grey matter ROIs as smoothing extent increased (Table 4, values in parentheses). Comparing the region-constrained analysis results, we observed a statistically significant (voxel-based thresholding, 5% FWER correction) increase in t-statistics across all grey matter ROIs as smoothing extent increased (Table 4; * and † = statistically significant difference; * = 3 > 0 mm, † = 5 > 3 mm). The group-level ROI mean t-statistics results were slightly more variable: there was a consistent increase in mean t-statistic comparing 3 mm smoothing to no smoothing, but a 50/50 split in ROIs in which mean t-statistics increased when comparing 3 mm–5 mm smoothing. These ROI and region-constrained results suggest that increasing the spatial smoothing extent tended to increase t-statistics overall by “smearing” activity.

**Table 4:** Comparison of the effects of spatial smoothing on t-statistics from the GLM output and significantly activated voxel counts for all 15 subjects. T-statistics: as spatial smoothing extent increased, peak t-statistics (in parentheses) decreased and statistically significant (voxel-based thresholding, 5% FWER corrected) increases in t-statistics (* and † symbols) were observed across all grey matter ROIs. The mean t-statistic in all ROI increased after 3 mm smoothing compared to no smoothing, and increased in PoCG and thalamus after 5 mm smoothing compared to 3 mm # active voxels and % white matter: as smoothing extent increased, the number of statistically significant voxels increased, measured using three thresholding approaches. Voxel is FDR corrected; cluster and TFCE are FWER corrected. ACC = anterior cingulate cortex; PoCG = postcentral gyrus; TFCE = threshold free cluster enhancement; * and † = statistically significant difference.

		0 mm FWHM	3 mm FWHM	5 mm FWHM
T-statistics	ACC	1.956 (5.392)	2.104* (4.030)	2.066 (3.102)
	Insula	2.674 (6.222)	2.977* (5.224)	2.968 (4.317)
	PoCG	2.423 (8.778)	2.959* (6.987)	3.265 (5.874)
	Thalamus	1.928 (7.467)	2.565* (6.797)	2.927 (5.389)

# active voxels	Voxel	14,484	66,984	140,389
	Cluster	48,332	82,837	109, 137
	TFCE	25,226	66,756	93,852

% white matter	Voxel	13,353	17.598	21.309
	Cluster	4.616	5.400	7.192
	TFCE	2.319	4.687	6.148

**Fig. 10:**
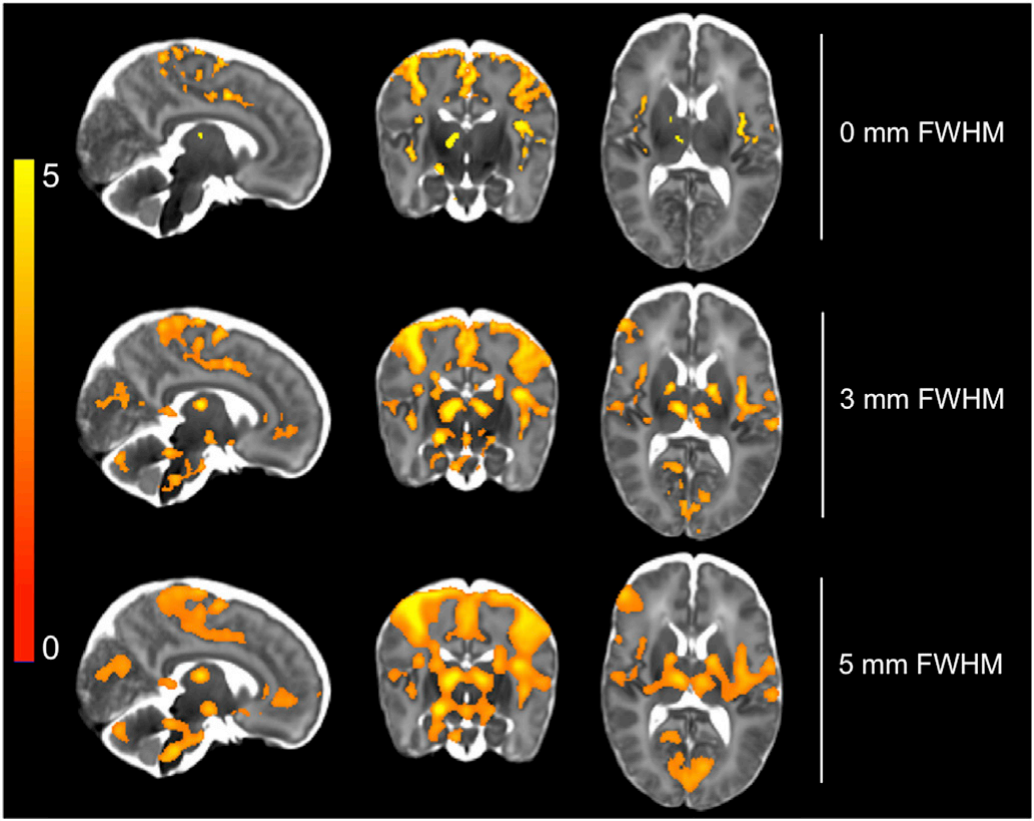
Comparison of the effects of spatial smoothing on t-statistics from the GLM output for all 15 subjects. Comparing thresholded (TFCE, default parameters, 5% FWER corrected) group activity maps, as spatial smoothing extent increased, the signal sensitivity increased in all ROIs, but the spatial specificity decreased. Not using spatial smoothing resulted in a lack of signal sensitivity in both cortical and subcortical structures. Using the 5 mm FWHM kernel, significant activity was incorrectly localised to non-grey matter regions, and distinct clusters of activity fused into massive clusters spanning several brain regions. Note, this spatial smoothing comparison was assessed using dHCP pipeline outputs only.

Comparing the number of significantly active voxels and the proportion of significant activity incorrectly localised to white matter, all three thresholding approaches demonstrated increasing number of active voxels and proportion of mislocalised activity with increasing smoothing extent (Table 4). Using the thresholded activity maps to qualitatively compare smoothing extents, a clear shift in the balance between spatial specificity and sensitivity to signal was visible, consistent with the quantitative t-statistic and voxel count comparisons (Fig. 10). Without smoothing, activations in many regions were tightly localised to grey matter, and as smoothing increased, activations became increasingly blurred across functionally distinct brain areas. However, not smoothing resulted in a thresholded activity map lacking several functionally important regions, such as ipsilateral thalamus, ACC, hippocampus, brainstem, and cerebellum. The minimal smoothing extent of 3 mm (1.5 times voxel size) appeared to be a reasonable compromise between gaining sensitivity to signal at the cost of spatial specificity.

### 3.5 Assessment of haemodynamic response function modelling

**Fig. 11:**
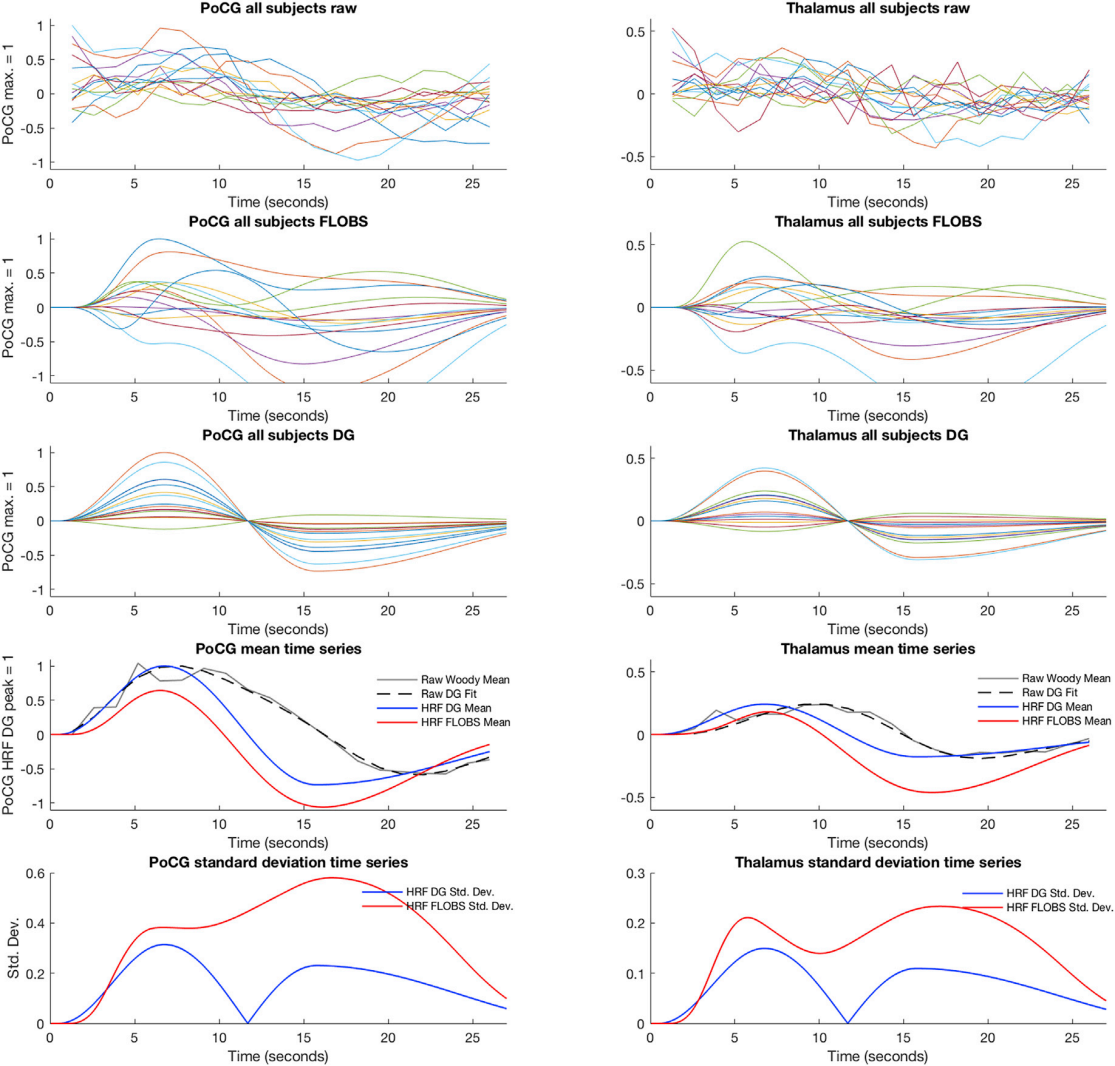
Peristimulus time plot analysis. The left column contains results for the postcentral gyrus (PoCG) ROI; the right column contains results for the thalamus (Thalamus) ROI. For all plots, the x-axis is time in seconds and displays a time window from t = 0 s (time of stimulus delivery) to t = 26 s. The y-axis for rows 1–4 are arbitrarily scaled so that the maximum value, as indicated on each y-axis, has a value of 1. Row 5 y-axis is in arbitrary units. Rows 1–3: peristimulus time plots for all individual subjects, averaged over all voxels in the region and all trials, using the raw data (Row 1), the three basis-function (FLOBS) HRF (Row 2), and the double gamma (DG) HRF (Row 3). Row 4: group mean time series plots. The raw data plots are arbitrarily scaled so that the double gamma function fit to the PoCG raw data has a maximum of 1. In both the PoCG and Thalamus, the DG HRF has a larger upshoot amplitude and a smaller undershoot amplitude compared to the FLOBS HRF. There are also differences in latencies to peak, as described in the main text. Row 5: group standard deviation time series plots. In both the PoCG and Thalamus, the FLOBS HRF has larger cross-subject variability, most noticeably during the undershoot component of the BOLD response. Note, for the mean and standard deviation plots in Rows 4–5, the values at each time point are calculated from the range of values across subjects at each time point for the DG and FLOBS responses displayed in Rows 2–3. Thus, the differences in mean and standard deviation between the DG and FLOBS models are visible prior to deriving a size statistic (2-norm) for the FLOBS model.

We assessed the effects of three HRF models on sensitivity to signal using the dHCP pipeline results. To explore the causes of variability in HRF model fits, we compared peristimulus time plots at both the subject and group levels in the PoCG and thalamus ROIs (Fig. 11). In the raw data, a considerable undershoot was visible (Fig. 11 Row 4, grey and black plots). Due to this undershoot not being modelled by the SG HRF, we excluded the SG from further peristimulus time series comparisons. The FLOBS model appeared to over-fit the data compared to the DG model. At the subject level, the fitted FLOBS HRFs exhibited a wide array of BOLD response shapes, including some biologically unlikely profiles (Fig. 11 Row 2). The reduced flexibility of the DG HRF appeared to make this model more robust to noise (Fig. 11 Row 3). To quantify these differences, we compared the baseline-to-peak latency and upshoot-to-undershoot ratio of the group mean DG and FLOBS HRFs to a double gamma function fit to the raw data group average (Fig. 11 Row 4). In both ROIs, the DG HRF had an upshoot-to-undershoot ratio more closely resembling the raw data. In the PoCG, the DG HRF had a latency to peak more closely resembling the raw data than the FLOBS HRF, but in the thalamus, this relationship was reversed. Comparing the between-subject variability in HRFs (measured as standard deviation), the FLOBS HRF had noticeably larger variability than the DG HRF, especially during the undershoot component of the BOLD response (Fig. 11 Row 5). Together these results suggest the SG HRF is an overly simple model that under-fits the BOLD response. The FLOBS model may be too flexible for the level of noise in infant fMRI data, resulting in over-fitting and large between-subject variability. The DG appeared to be a reasonable compromise between under-fitting and over-fitting of the modelled response to the data.

Comparing the group-level ROI analysis results, we found the DG HRF produced the largest mean t-statistics in all grey matter ROIs compared to both the SG and FLOBS HRFs (Table 5). The constrained-region group comparisons of HRF models demonstrated statistically significantly (voxel-based thresholding, 5% FWER corrected) larger t-statistics in the DG HRF compared to the SG, localised to the insula. There were no statistically significant differences in t-statistics between the DG and FLOBS models. Similarly, the DG HRF resulted in the largest number of active voxels compared to both SG and FLOBS models using all three thresholding approaches (Table 5). Using the thresholded group activity maps to qualitatively compare HRF models, the DG HRF produced noticeably increased activity in both cortical and subcortical structures (Fig. 12). Overall, these GLM-based results were in line with our peristimulus time series results, and suggested the DG HRF model provides greatest sensitivity to signal compared to both the SG and FLOBS models.

**Table 5:** Comparison of the effects of HRF modelling on t-statistics from the GLM output and significantly activated voxel counts for all 15 subjects. T-statistics: the double gamma HRF had greatest mean t-statistic in all grey matter ROIs (maximum t-statistic in parentheses). # active voxels: the double gamma HRF had the largest number of significantly activated voxels using all three thresholding approaches. Voxel is FDR corrected; cluster and TFCE are FWER corrected. ACC = anterior cingulate cortex; PoCG = postcentral gyrus; TFCE = threshold free cluster enhancement; * = statistically significant difference.

		Single gamma	Double gamma	FLOBS
T-statistics	ACC	1.870 (4.889)	2.104 (4.030)	1.905 (3.953)
	Insula	2.773 (5.269)	2.977* (5.224)	2.141 (4.400)
	PoCG	2.877 (7.182)	2.959 (6.987)	2.729 (5.824)
	Thalamus	1.985 (5.709)	2.565 (6.797)	2.366 (5.920

# active voxels	Voxel	33.421	66,984	57,569
	Cluster	72,987	82,837	46,648
	TFCE	38,896	66,756	29,665

**Fig. 12:**
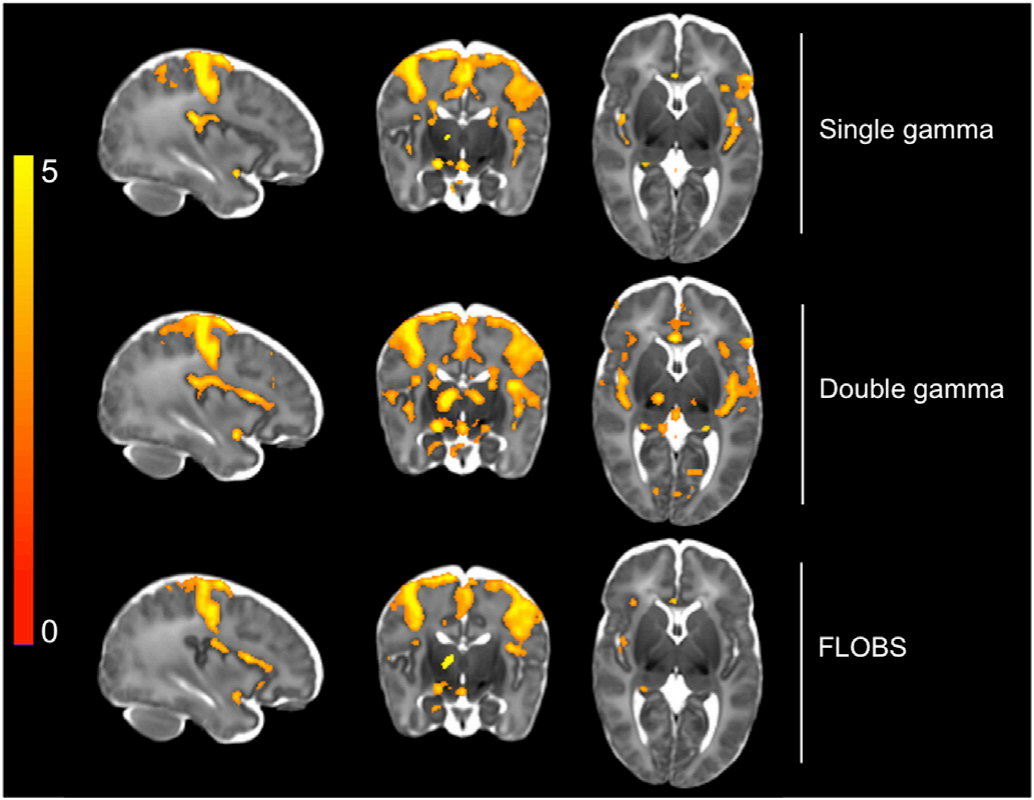
Comparison of the effects of HRF modelling on t-statistics from the GLM output for all 15 subjects. Comparing thresholded (TFCE, default parameters, 5% FWER corrected) group activity maps, the double gamma HRF had greatest sensitivity to signal in both cortical and subcortical structures, most noticeable in ipsilateral thalamus.

### 3.6 Overall effects of the dHCP pipeline on statistical results

Finally, we compared the overall effect of our FEAT and dHCP pipelines on GLM results. To generate the group-level results, both pipelines included FIX denoising, 3 mm FWHM spatial smoothing, and the neonatal DG HRF at the subject-level. Comparing the mean t-statistics of the ROI analysis, the dHCP pipeline resulted in increased mean t-statistics in all grey matter ROIs (Table 6). The constrained-region group comparisons demonstrated statistically significantly (voxel-based thresholding, 5% FWER corrected) larger t-statistics in the dHCP pipeline compared to the FEAT pipeline, localised to the PoCG. This is in contrast to our assessments of MCDC and spatial normalisation above (sections 3.1*.* and 3.2.), where we did not observe statistically significant differences in t-statistics when contrasting these pipeline differences in isolation.

**Table 6:** Overall comparison of FEAT and dHCP pipelines using t-statistics from the GLM output and significantly activated voxel counts for all 15 subjects. T-statistics: using the dHCP pipeline, all grey matter ROIs showed an increase in mean t-statistic (maximum t-statistic in parentheses). This increase in t-statistics was revealed to be statistically significant (voxel-based thresholding, 5% FWER corrected) using the constrained-region analysis, localised to the PoCG. # active voxels: the dHCP pipeline also resulted in an increase in the number of significantly active voxels using all three thresholding approaches. % white matter: voxel-based thresholding was not valid due to zero voxels being activated. Cluster-based thresholding showed a clear reduction in mislocalised activity using the dHCP pipeline. TFCE-based thresholding showed a very modest increase in mislocalised activity using the dHCP pipeline, possibly due to unavoidable ‘enhancement’ of white matter voxels by the neighbouring grey matter. Voxel is FDR corrected; cluster and TFCE are FWER corrected. ACC = anterior cingulate cortex; PoCG = postcentral gyrus; TFCE = threshold free cluster enhancement; * = statistically significant difference.

		FEAT	dHCP
T-statistics	ACC	1.529 (3.518)	2.104 (4.030)
	Insula	2.651 (5.244)	2.977 (5.224)
	PoCG	2.822 (6.568)	2.959* (6.987)
	Thalamus	2.168 (6.197)	2.565 (6.797)

# active voxels	Voxel	0	66,984
	Cluster	60,175	82,837
	TFCE	16,823	66,756

% white matter	Voxel	–	17.598
	Cluster	10.051	5.400
	TFCE	4.412	4.687

**Fig. 13:**
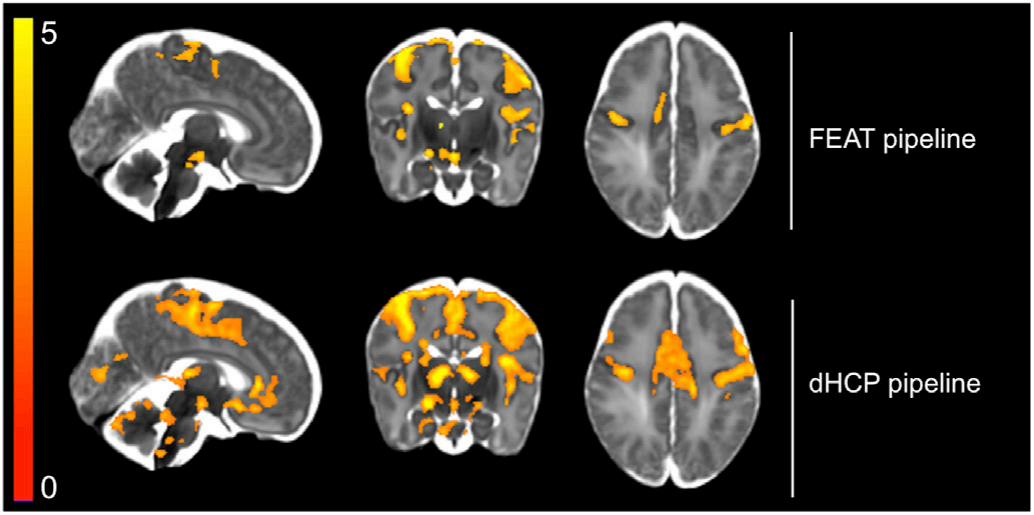
Overall comparison of FEAT and dHCP pipelines using t-statistics from the GLM output for all 15 subjects. Comparing the thresholded group activity maps (TFCE, default parameters, 5% FWER corrected), the dHCP pipeline resulted in increased sensitivity to signal in both cortical and subcortical regions. Compared to the unilateral activity detected in the thalamus and cingulate cortex using the FEAT pipeline, the bilateral activity detected in these regions using the dHCP pipeline could lead to a substantially different interpretation of the data.

Similarly to the mean t-statistic results, the dHCP pipeline resulted in a larger number of significantly active voxels compared to the FEAT pipeline using all three thresholding approaches (Table 6). The different thresholding approaches were less clear-cut in quantifying the proportion of activity mislocalised to white matter. Voxel-based thresholding was not appropriate here, because no activation survived thresholding with the FEAT pipeline. Cluster-based thresholding suggested a clear improvement in spatial specificity using the dHCP pipeline. TFCE thresholding revealed almost identical levels of mislocalised activity in both pipelines. However, given our assessment of spatial normalisation (section *3.2.*) clearly demonstrated improved alignments using the dHCP pipeline, the similar proportions of activity in white matter measured here appear to be a TFCE “artefact”. That is, given the increase in sensitivity to signal seen with the dHCP pipeline, we would expect this substantially larger grey matter activity to unavoidably ‘enhance’ neighbouring white matter t-statistics. Finally, using the thresholded group activity maps to qualitatively compare pipelines, the dHCP pipeline demonstrated noticeably increased activity in both cortical and subcortical structures (Fig. 13). Taken together, these quantitative and qualitative GLM-based pipeline comparisons revealed dramatically improved spatial specificity and sensitivity to signal using the dHCP pipeline.

## 4 Discussion

In this work, we report and validate an extension of the dHCP fMRI preprocessing pipeline for the analysis of evoked brain responses in infants. We identify optimal processing choices and show that the extended dHCP pipeline substantially improves sensitivity to signal and spatial specificity of activity detected in response to a noxious stimulus. There are several advantages to optimising the dHCP pipeline to characterise noxious-evoked BOLD activity. In contrast to resting-state data, the time course of the signal of interest is determined by the experimenter, meaning that model parameters and statistics can be assessed using a general linear modelling approach (Boynton et al., 2012). As noxious stimulation activates a large array of distinct brain areas in infants (Goksan et al., 2015; Williams et al., 2015), it provides multiple discrete regions in which model parameters and statistics can be examined. In addition, reflex limb withdrawal in infants is often evoked by low-intensity noxious stimuli (Hartley et al., 2015), providing an opportunity to examine whether stimulus-correlated motion artefacts can be effectively minimised.

Subject motion and large inter-subject variability in brain morphology represent two substantial challenges in the analysis of infant MRI data. We show that slice-to-volume motion correction (Andersson et al., 2017) and estimated dynamic distortion correction (Andersson et al., 2018) dramatically reduce the effects of head motion, increasing sensitivity to BOLD responses across the brain. While these results are compelling, it should be noted that EDDY, which was used for both slice-to-volume and dynamic distortion correction, was designed for diffusion data, and its use on functional data is undocumented and not officially supported by FSL. For fMRI, EDDY uses a predictive model in which each volume is treated as if it were a diffusion B0 image. The results herein, and other results in preparation (not shown), are starting to build a strong case for the use of EDDY on fMRI data.

Another major challenge is to ensure that functional data from infants is accurately registered to a standard template to facilitate group analysis. Accurately computing these spatial normalisation transformations is challenging because of the rapid developmental changes in brain size, volume, and gyrification in infants (Dubois et al., 2014; Dubois and Dehaene-Lambertz, 2015). The dHCP pipeline uses infant-appropriate boundary-based registration (BBR) parameters for the functional-to-structural registration that account for the inversion in grey-matter/white-matter contrast compared to adults, and an advanced registration tool (ANTs's Syn) for the structural-to-standard registration that uses multiple warp resolutions among other optimisations. This leads to significant improvements in the alignment of functional images to the standard infant template, throughout the brain, especially at the cortical surface, the brainstem, and the cerebellum. Overall, there is a substantial benefit in using optimised spatial normalisation.

FIX denoising is a semi-automatic spatial ICA-based cleanup approach used to remove noise artefacts from fMRI data, and has been successfully implemented in adult and infant fMRI data to greatly improve sensitivity to signal (Ball et al., 2016; Griffanti et al., 2014; Salimi-Khorshidi et al., 2014). To date, there has been no formal assessment of the benefits of FIX denoising applied to neonatal fMRI data. We found the use of FIX denoising resulted in a dramatic increase in the number of significantly active voxels, and improved signal detection across multiple cortical and subcortical brain regions, at both the subject and group levels. FIX denoising allowed us to obtain good quality data from participants that would otherwise have been rejected due to artefacts. Fig. 8 demonstrates the dramatic improvement FIX denoising can have on the spatial properties of a single subject's activity map: with FIX denoising performed, the relatively smooth activity map with large activations well localised to grey matter regions is strongly suggestive of improved modelling of neural responses rather than motion. It is clear that effective denoising is a crucial element of neonatal fMRI analysis and should be incorporated into analysis pipelines.

Noxious-evoked BOLD activity in the infant is generated in a multitude of brain regions, from small grey matter nuclei to the entire primary somatomotor cortex (Goksan et al., 2015; Williams et al., 2015). Thus, the noxious stimulation used in this experiment provided an opportunity to investigate the effect of spatial smoothing (Lowe and Sorenson, 1997). Smoothing increases SNR and improves between-subject anatomical overlap of the functional data in standard space, but can result in poorer spatial specificity. We demonstrated that omitting spatial smoothing produced a substantial drop in sensitivity to signal in ACC, thalamus, hippocampus, brainstem, cerebellum, as well as other cortical and subcortical regions. The use of a smoothing kernel more than twice the voxel size (i.e. the 5 mm smoothing kernel) resulted in increased sensitivity to some signal at the significant cost of spatial specificity, with a large increase in the proportion of activity incorrectly localised to white matter and the fusion of clusters that were clearly distinct with reduced spatial smoothing. The fusion of significantly active regions that span multiple functionally distinct brain areas is problematic when exploring noxious-evoked brain activity, due to the close proximity of several distinct brain areas where noxious-evoked brain activity is generated (Woo et al., 2014). This is exemplified in the perisylvian/operculoinsular region, where the lateral-most region of area SI of the postcentral gyrus is adjacent to area SII in the parietal operculum, which itself is adjacent to the posterior insula, regions all typically involved in processing noxious stimuli in both adults and infants (Apkarian et al., 2005; Goksan et al., 2015; Tracey and Mantyh, 2007; Williams et al., 2015). To explore and understand the role of each of these distinct brain regions, we must avoid artificially combining and blurring the signal recorded across discrete brain regions. In practice, this requires avoiding large spatial filters that could easily cover multiple infant brain regions, and lead to a lack of spatial specificity and problematic spatial inference that would dramatically affect the interpretation of activity maps. Minimal smoothing of 1.5 times the voxel size yielded an optimal balance of spatial specificity and sensitivity to signal in our data. It must be noted that all our analyses were performed in volumetric space, so these conclusions would not apply to surface-based analyses. The assessment of optimal spatial smoothing extent will have to be investigated in future surface-based work.

In general, the researcher's decision about spatial smoothing needs to be based on two things: first, data quality and quantity, and second, on the specific research question. Regarding data quality and quantity, smoothing the data involves a trade-off between increasing smoothing to increase SNR and statistical power with the undesirable effect of blurring your image and reducing spatial specificity. Spatial smoothing might not be needed if SNR is reasonably good or if a large dataset is available. In general, the less smoothing the better, but it is a trade-off. Regarding the research question, if you are interested in exploring small brain regions or short-range functional connectivity, then spatial smoothing may have to be omitted. And conversely, if you are exploring larger regions or functional connectivity between distantly separated regions, then smoothing can reasonably be done if an increase in statistical power is needed. Our findings emphasise the point that if spatial smoothing is performed, the extent should be limited as much as possible due to the unwanted effect of blurring across tissue-type boundaries and functional-region boundaries, an effect which is greater in infants than adults due to the much smaller brain volume.

The immaturity of the infant neurodynamic and haemodynamic responses, and their coupling, suggests that the adult canonical HRF is inappropriate, and adoption of a neonate-specific HRF function would improve the accuracy of modelling infant haemodynamic brain activity (Arichi et al., 2012; Colonnese et al., 2008). Both near-infrared spectroscopy and BOLD fMRI studies demonstrate that the haemodynamic response has a longer latency to peak in infants as compared with adults (Arichi et al., 2012; Roche-Labarbe et al., 2014; Slater et al., 2006). Arichi and colleagues characterised the infants' BOLD response to a somatosensory stimulus using a double gamma HRF and, in term infants, estimated the latency to peak to be approximately 7 s and the ratio of upshoot-to-undershoot to be approximately 1:1 (Arichi et al., 2012). We found the infant double gamma HRF had the greatest sensitivity to signal compared to the single gamma and FLOBS HRF models. Using the infant double gamma function, we were able to detect robust signal in multiple cortical and subcortical brain regions typically included in descriptions of adult pain networks (Apkarian et al., 2005; Tracey and Mantyh, 2007), and as previously reported in infants using independent datasets (Goksan et al., 2015; Williams et al., 2015). We observed large undershoots in our data, making the single gamma function an inappropriate choice, as it fails to model the undershoot leading to model under-fitting. Interestingly, the FLOBS HRF model had lower-than-expected sensitivity to signal, which appeared to be due to model over-fitting. Compared to the double gamma HRF, the increased flexibility of the FLOBS HRF resulted in a large increase in between-subject variability in modelled BOLD response morphology. We observed differences in latency to peak between postcentral gyrus and somatosensory thalamus, and in both ROIs, there was substantial cross-subject variability in undershoot morphology, which points to there being considerable scope for improved neonatal HRF estimation methods still. It is worth noting that the experimental stimulus used by Arichi and colleagues to develop their term neonatal HRF models was somatosensory (non-noxious). The HRF models were developed from a sample with an age-range similar to ours and from somatomotor regions. It is therefore plausible, due to the similarity of the experimental paradigms, that the double gamma function was ideally tuned to explain the bulk of the BOLD signal variance evoked by our noxious stimulus. This may have resulted in a type of ‘Goldilocks Effect’, whereby both simplifying the HRF model (i.e. using the single gamma HRF) and also increasing its complexity (i.e. using the FLOBS HRF) could have shifted the model away from a parameterisation that was ‘just right’. Our data might therefore not profit from the increased flexibility afforded by the FLOBS model. It is possible that data with a wider range of ages or different stimulus modalities may still benefit from the flexibility of using a basis set HRF model. Further exploration of this topic is still very much warranted.

It is important to note that several of our findings are not specific to the neonatal population. From the adult literature, we know the advantages of using FIX denoising (Griffanti et al., 2014), of using the BBR cost function (Greve and Fischl, 2009), and of ANT's SyN over FSL's FNIRT (Klein et al., 2009). Regarding motion and distortion correction, the advantages of volume-to-volume plus slice-to-volume motion correction over volume-to-volume motion correction alone (Andersson et al., 2017), and the advantages of estimated dynamic distortion correction over static distortion correction (Andersson et al., 2018), are also established in adult MRI. Here, we demonstrate that these advances in analysis provide significant improvements in neonatal fMRI data processing too. We also expect this pipeline to be useful for studies throughout childhood, and in other cohorts where head motion, and challenging variations within and across individuals exist. Of course, the neonate-specific standard templates and HRF models would have to change according to the population in question.

While the current study did not explore the optimisation of data acquisition protocols, the issue of data acquisition is also of central importance to the advancement of neonatal fMRI research quality. If you compare adult and infant data with identical spatial resolution (e.g. 2 mm isotropic voxels), the ‘effective resolution’ (the ability to distinguish neighbouring structures) is much lower in infants. Therefore, achieving good spatial resolution is particularly valuable in studies of neonates. Here, we have obtained a higher spatial resolution than in our previous studies (Goksan et al., 2018, 2015) through the use of multiband imaging (Moeller et al., 2010; Xu et al., 2013) and an echo time optimised for neonates (Goksan et al., 2017). However, severe trade offs are faced when increasing resolution. Increasing spatial resolution will dramatically decrease SNR, which decreases proportionally to voxel volume. Further, increasing the multiband factor - to maintain an acceptable repetition time - also reduces SNR, particularly in subcortical regions. Maintaining SNR is particularly important in infants, as their BOLD signal is intrinsically low, compared to adults (Arichi et al., 2012). We felt that the current acquisition provided a good balance of resolution and SNR, but more detailed assessment of this trade off, and use of infant-specific head coils, would be of great interest (Hughes et al., 2017).

## 5 Conclusion

In summary, we successfully adapted and optimised an extended version of the dHCP fMRI preprocessing pipeline to an infant stimulus-based fMRI dataset, which in this case was a mild noxious stimulus applied to the infant's foot. We assessed the effects of implementing this pipeline on spatial specificity and sensitivity to signal, comparing the results to a more traditional FSL FEAT-based pipeline, to ensure that the dHCP pipeline's more extensive data manipulation and signal variance reduction did not inadvertently remove signal-of- interest. The dHCP pipeline's sophisticated motion correction, distortion correction, and spatial normalisation steps provided dramatic improvements in both sensitivity to signal and spatial specificity, measured using a range of independent, quantitative metrics. We examined the effect of FIX denoising, spatial smoothing, and HRF modelling on both subject-level and group-level results, and found that the data cleanup provided by FIX, with minimal spatial smoothing, and an age-appropriate double gamma HRF, resulted in the optimal outcomes. These improvements were detectable at both the subject and group level, and both before and after GLM modelling. Importantly, we demonstrate that the dHCP preprocessing pipeline can be adapted for use on stimulus-based functional data, not just resting-state data for which the pipeline was initially being developed. It is also noteworthy that our dataset is independent of the dHCP, acquired with a different data acquisition protocol, thus highlighting the flexibility of this now-generalised preprocessing pipeline. Adoption of standardised, optimised analysis methods will improve infant fMRI data interpretation, minimise the heterogeneity in fMRI analysis, and facilitate comparison across studies. As the fMRI field moves from group-level analysis to subject-level analysis (Finn et al., 2015; Tavor et al., 2016; Vogt, 2015), the optimisation of data preprocessing for the infant population is imperative. The demonstration that the dHCP analysis pipeline can be successfully implemented to measure complex noxious-evoked haemodynamic activity in the infant brain is a valuable advance for the field of neonatal neuroimaging. This work outlines the foundations on which further infant fMRI research can be conducted, and provides a platform to address fundamental neuroscientific questions, such as investigating how environmental factors shape central nervous system function during early human development.

## Conflicts of interest

MJ, SS, and JA receive royalties from the commercial licensing of FSL (it is free for non-commercial use). The authors report no other conflicts of interest.
