# Supplementary material for: Optimising neonatal fMRI data analysis: Design and validation of an extended dHCP preprocessing pipeline to characterise noxious-evoked brain activity in infants
Source: Neuroimage. 2019 Feb 1;186:286–300. doi: 10.1016/j.neuroimage.2018.11.006 (PMC6347570; doi:10.1016/j.neuroimage.2018.11.006)
Supplement: Multimedia component 1 [file mmc1.docx]

**Supplementary material**

**Example neonatal FIX components**

Here, we have included examples of spatial independent component analysis (sICA) components commonly seen in our data. These components are independent signals in the fMRI data that originate from independent signal-sources, eight of which we show below. For all figures, we present the spatial map of the component at several axial slices on the left upper pane, and at a single sagittal and coronal slice on the right upper pane. We also provide the component’s time series in the left lower pane, and the power spectrum of the time series in the right lower pane. All components are displayed using FSL’s FSLeyes.

The nature and origin of these neonatal sICA components are no different from that of adults, and we strongly recommend those intending on using FIX denoising in their own data to read the excellent “how-to” guide by Griffanti and colleagues (Griffanti et al., 2017). Our examples below are deliberately displayed and named to resemble the adult examples in Griffanti et al. (2017), as we hope this will greatly facilitate comparison.

It is important to note that at the spatial and temporal resolution of our data, it is not always possible to unequivocally identify the signal-source of a component. For this reason, we have labelled the component in Supp. Fig. 4 as ‘Cerebrospinal fluid pulsation (and arteries?)’. Similarly, we have included a summary head motion time series (red dotted time series) in the left lower pane of Supp. Fig. 2 and 7 to demonstrate that the subject’s head motion is clearly the signal-source for the ‘Motion artefact’ and ‘Multiband artefact’ components.


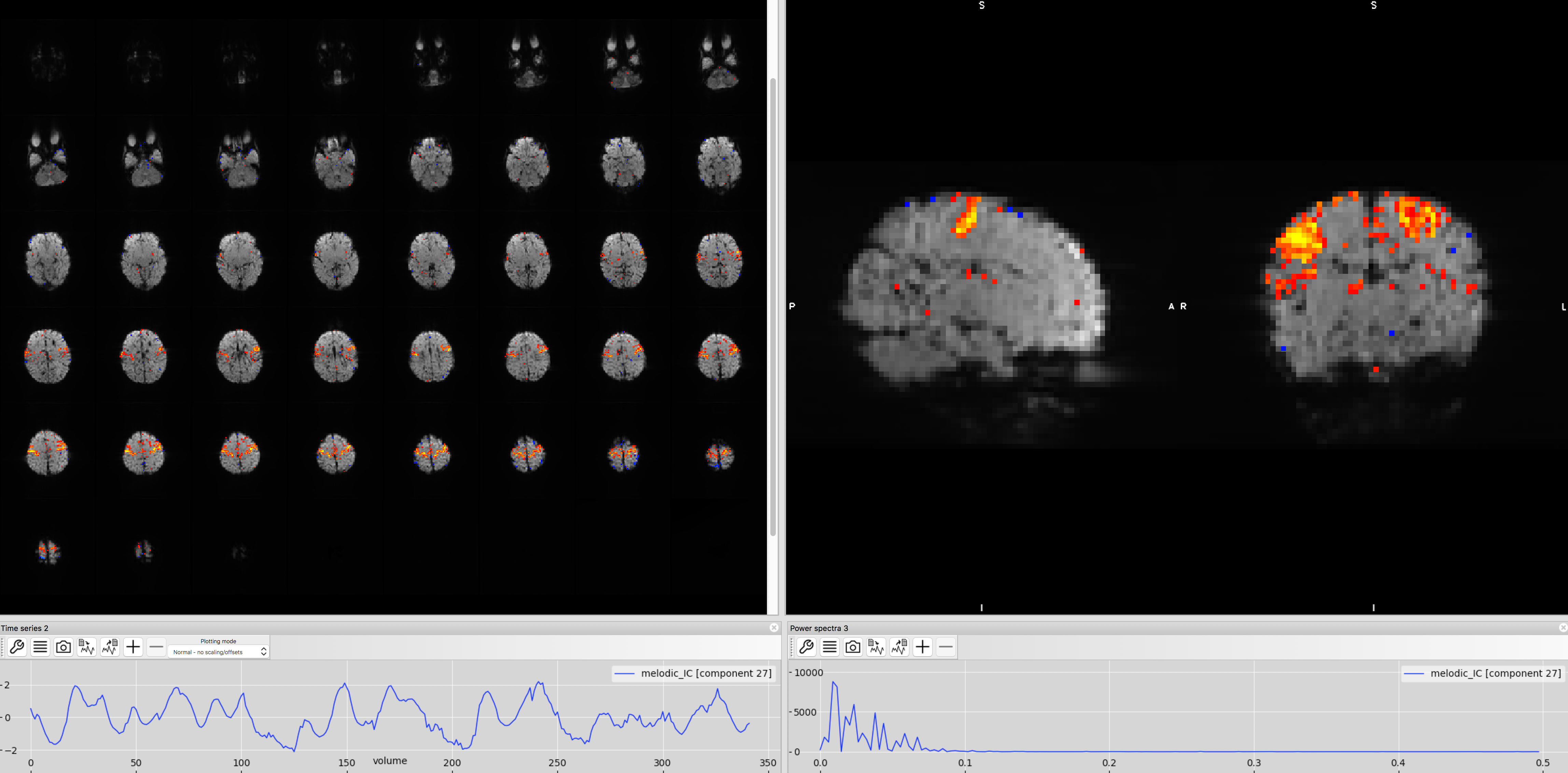


*Supp. Fig. 1: Signal.*


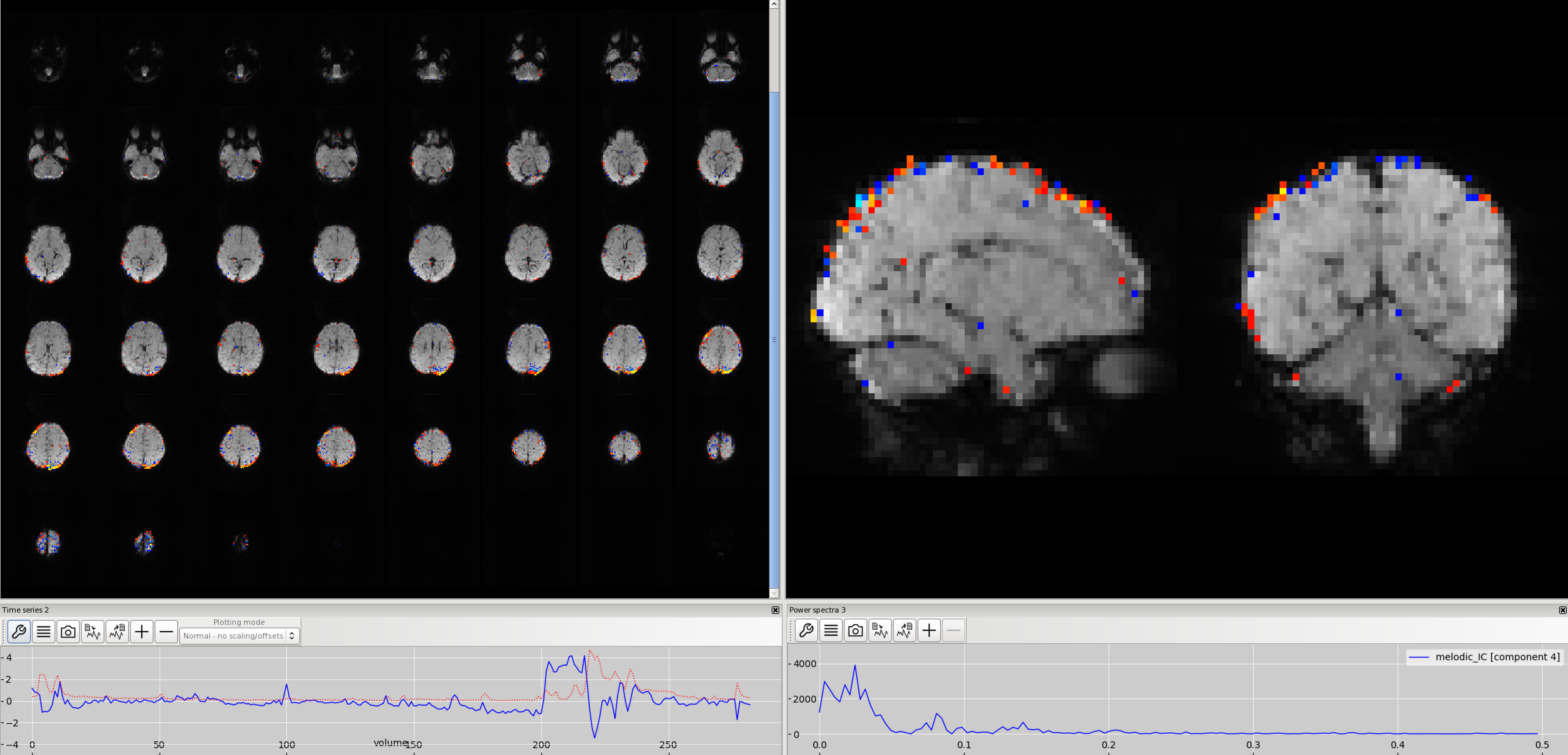


*Supp. Fig. 2: Motion artefact.*


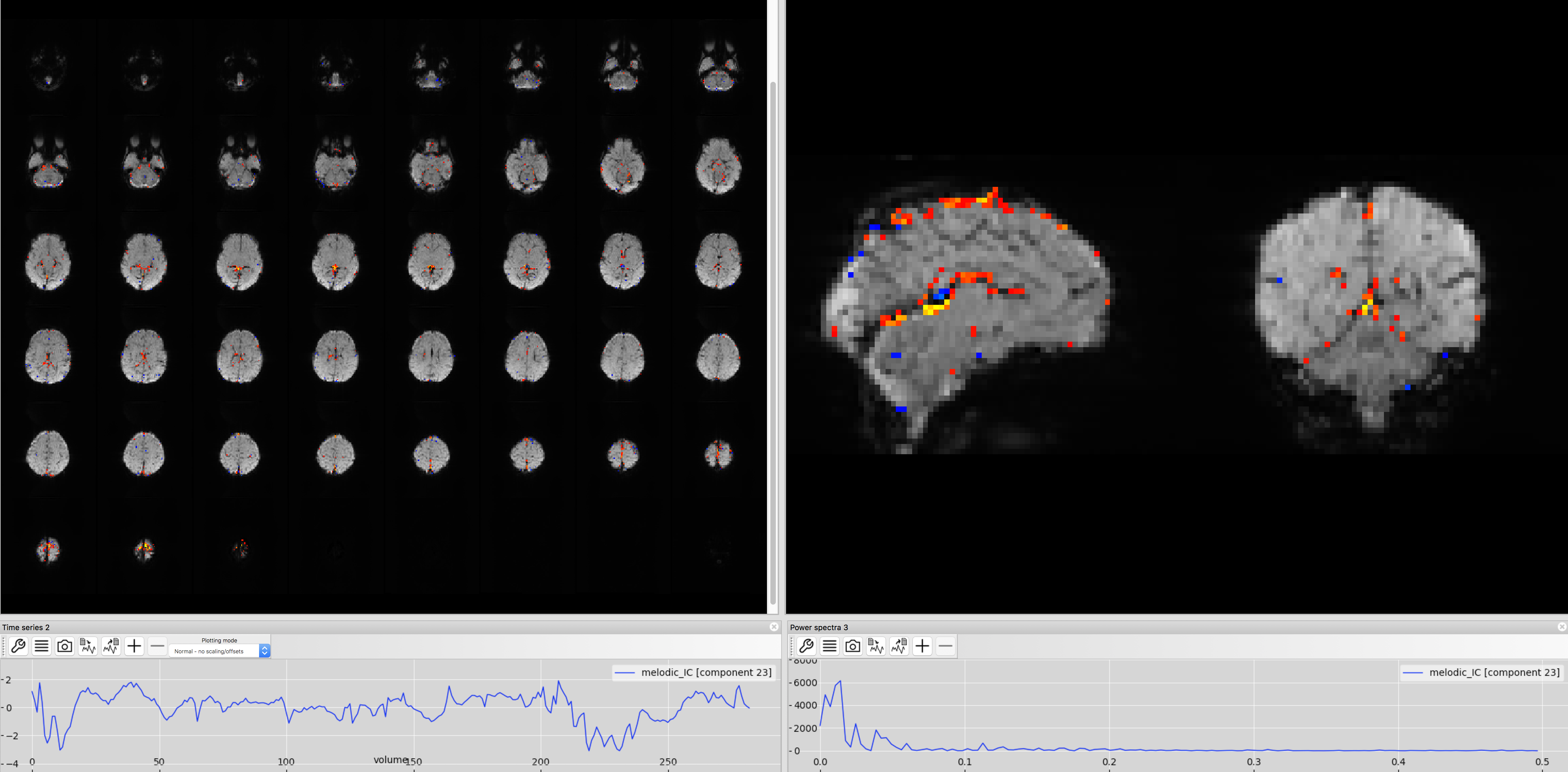


*Supp. Fig. 3: Vein.*


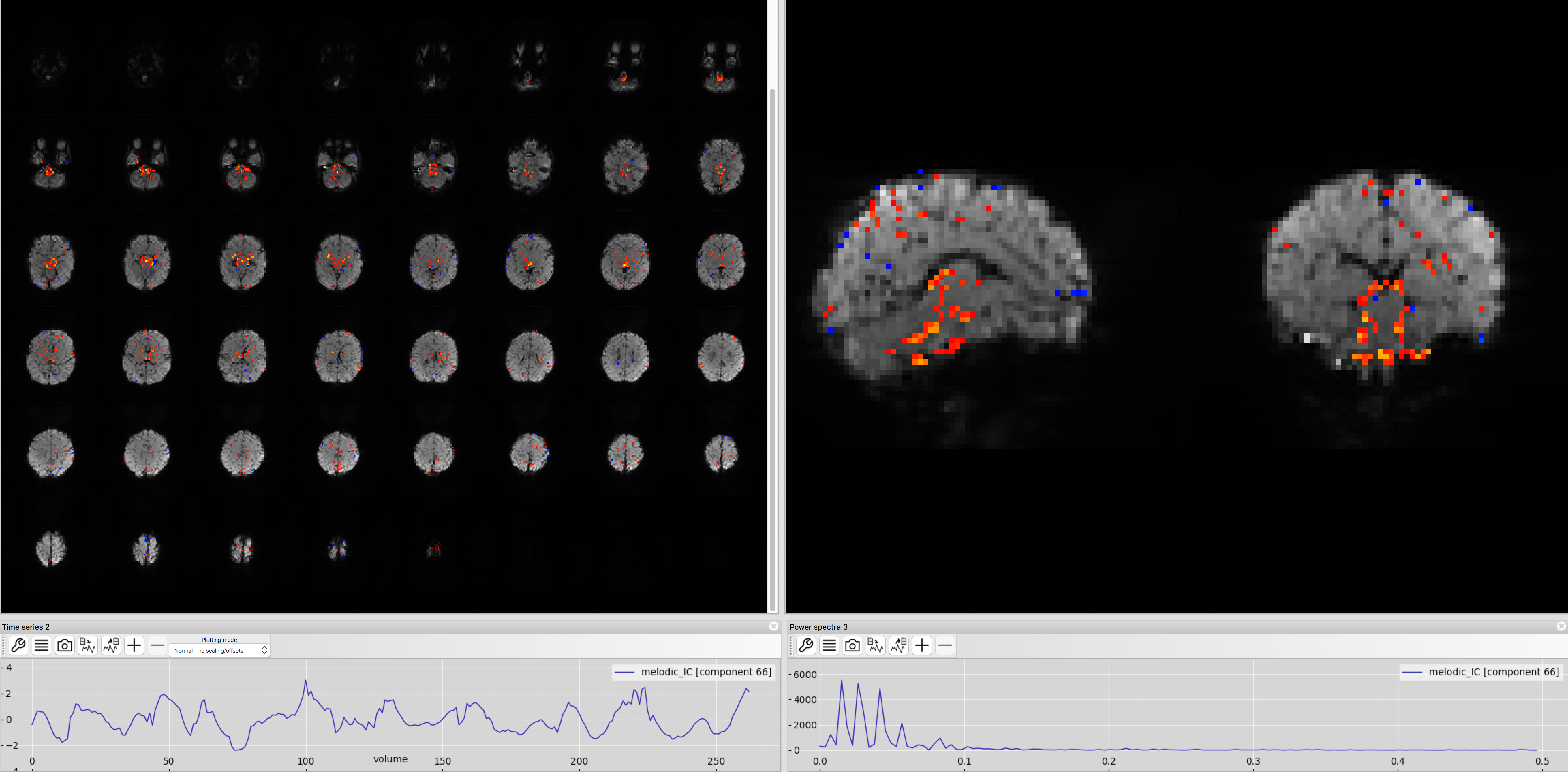


*Supp. Fig. 4: Cerebrospinal fluid pulsation (and arteries?).*


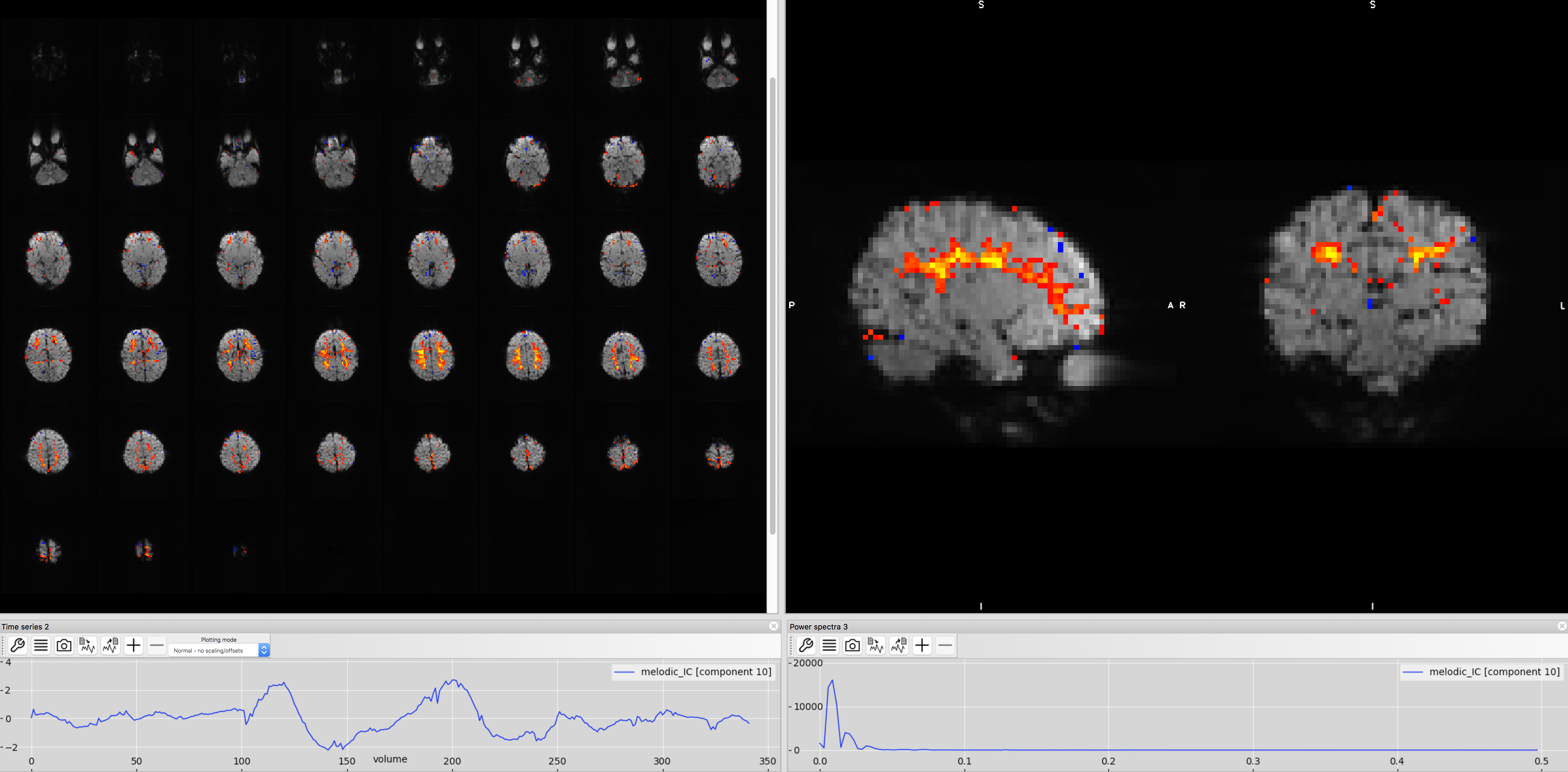


*Supp. Fig. 5: Fluctuations in subependymal (and transmedullary) veins.*


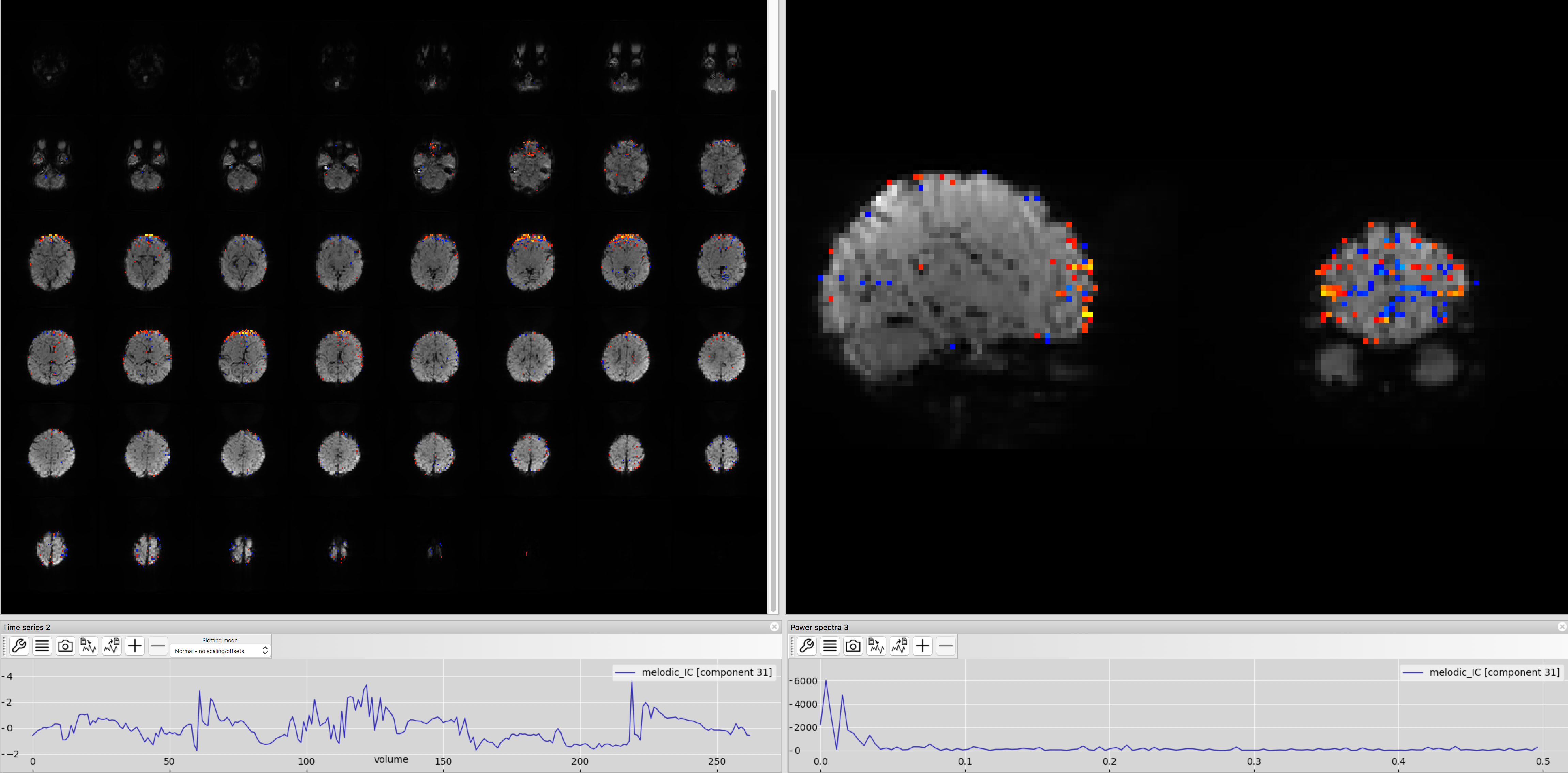


*Supp. Fig. 6: Susceptibility artefacts.*


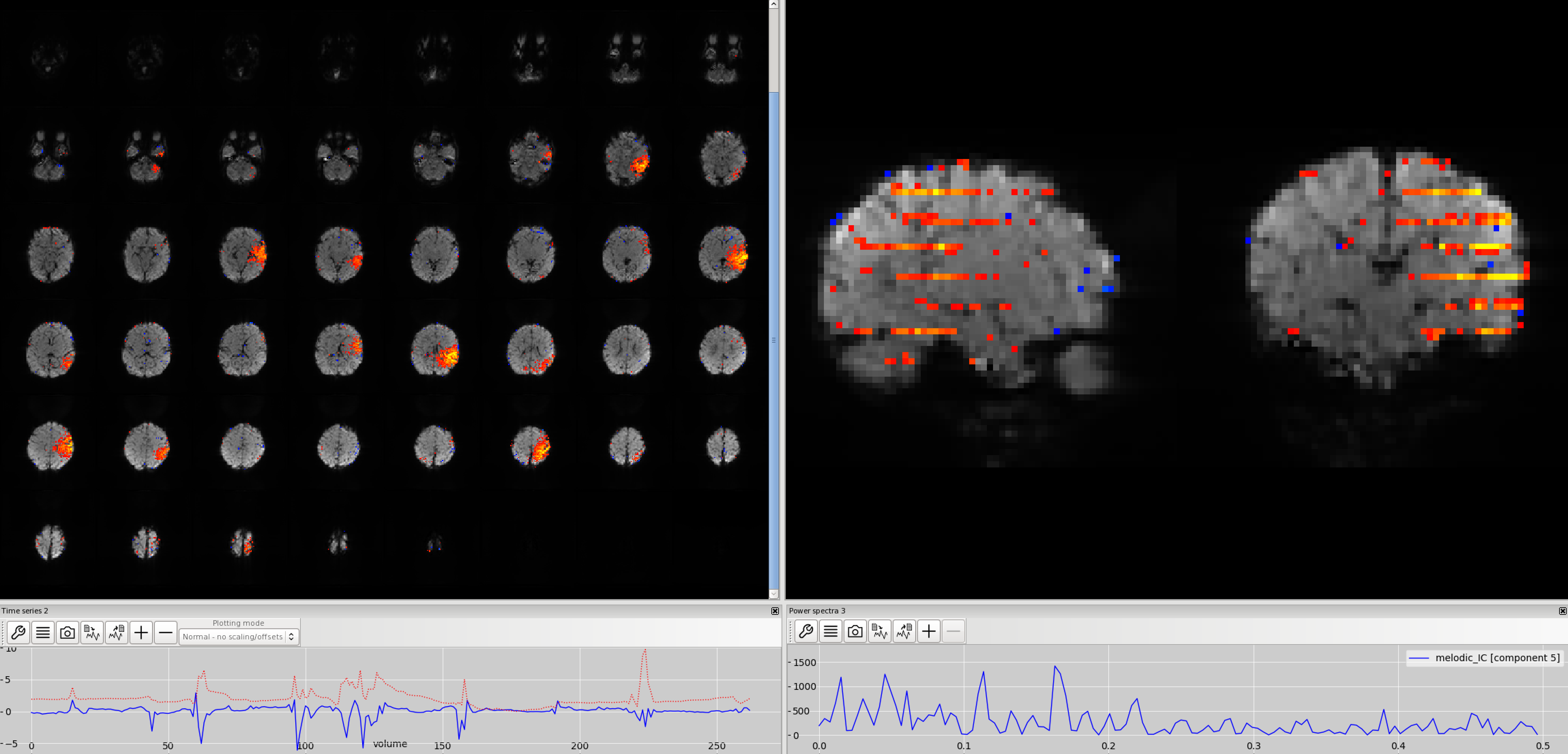


*Supp. Fig. 7: Multiband artefact.*


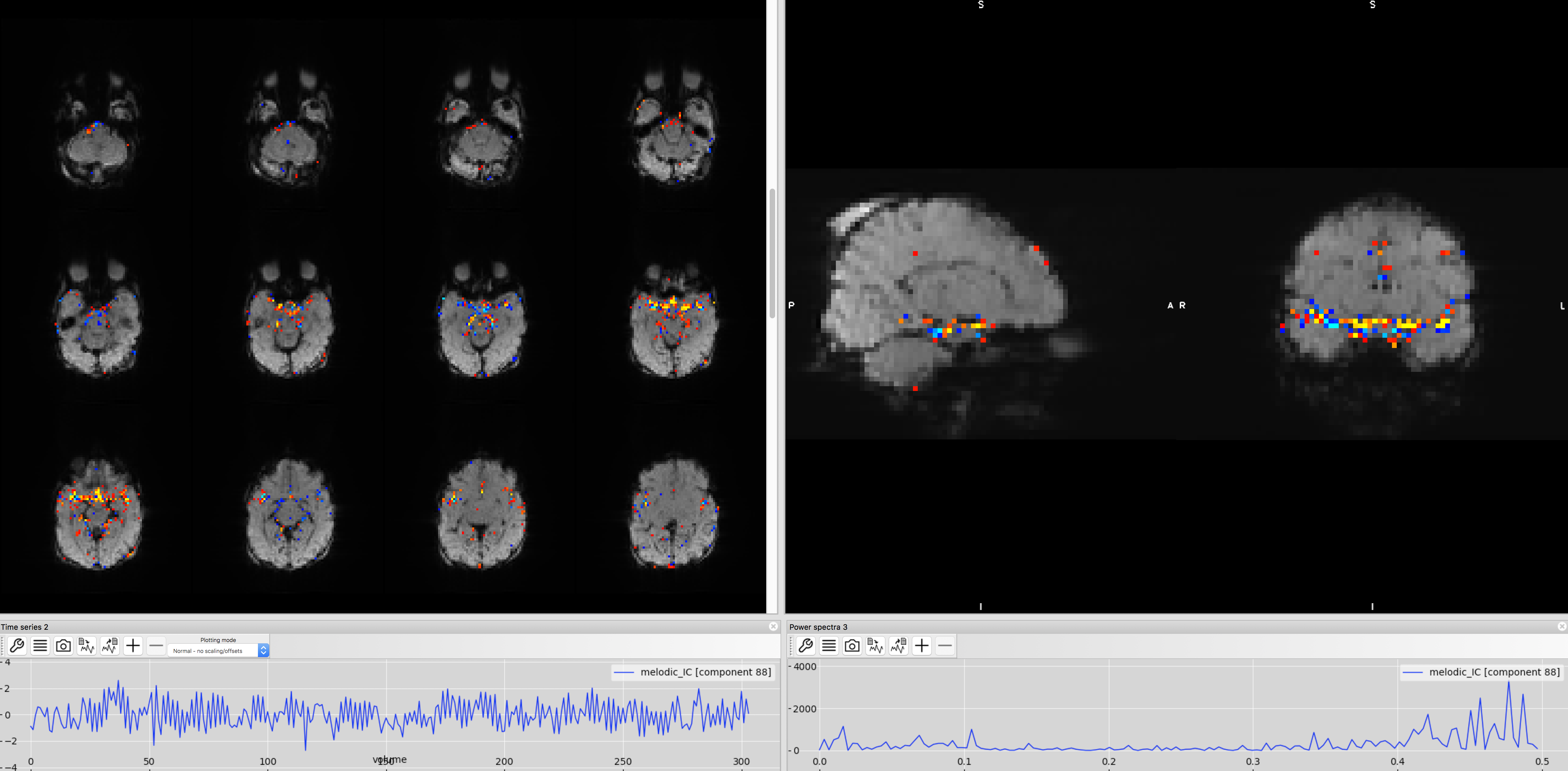


*Supp. Fig. 8: MRI-related artefact.*

**References**

Griffanti, L., Douaud, G., Bijsterbosch, J., Evangelisti, S., Alfaro-Almagro, F., Glasser, M.F., Duff, E.P., Fitzgibbon, S., Westphal, R., Carone, D., Beckmann, C.F., Smith, S.M., 2017. Hand classification of fMRI ICA noise components. NeuroImage, Cleaning up the fMRI time series: Mitigating noise with advanced acquisition and correction strategies 154, 188–205. https://doi.org/10.1016/j.neuroimage.2016.12.036
